# Cefixime removal via WO_3_/Co-ZIF nanocomposite using machine learning methods

**DOI:** 10.1038/s41598-024-64790-2

**Published:** 2024-06-15

**Authors:** Amir Sheikhmohammadi, Hassan Alamgholiloo, Mohammad Golaki, Parsa Khakzad, Esrafil Asgari, Faezeh Rahimlu

**Affiliations:** 1 Department of Environmental Health Engineering, School of Health, Khoy University of Medical Sciences, Khoy, Iran; 2 Student Research Committee, School of Health, Shiraz University of Medical Sciences, Shiraz, Iran; 3 Department of Environmental Health Engineering, School of Public Health, Zanjan University of Medical Sciences, Zanjan, Iran

**Keywords:** Cefixime, WO_3_/Co-ZIF, ANN, SVR, GA, Environmental sciences, Chemistry

## Abstract

In this research, an upgraded and environmentally friendly process involving WO_3_/Co-ZIF nanocomposite was used for the removal of Cefixime from the aqueous solutions. Intelligent decision-making was employed using various models including Support Vector Regression (SVR), Genetic Algorithm (GA), Artificial Neural Network (ANN), Simulation Optimization Language for Visualized Excel Results (SOLVER), and Response Surface Methodology (RSM). SVR, ANN, and RSM models were used for modeling and predicting results, while GA and SOLVER models were employed to achieve the optimal conditions for Cefixime degradation. The primary goal of applying different models was to achieve the best conditions with high accuracy in Cefixime degradation. Based on R analysis, the quadratic factorial model in RSM was selected as the best model, and the regression coefficients obtained from it were used to evaluate the performance of artificial intelligence models. According to the quadratic factorial model, interactions between pH and time, pH and catalyst amount, as well as reaction time and catalyst amount were identified as the most significant factors in predicting results. In a comparison between the different models based on Mean Absolute Error (MAE), Root Mean Square Error (RMSE), and Coefficient of Determination (R^2^ Score) indices, the SVR model was selected as the best model for the prediction of the results, with a higher R^2^ Score (0.98), and lower MAE (1.54) and RMSE (3.91) compared to the ANN model. Both ANN and SVR models identified pH as the most important parameter in the prediction of the results. According to the Genetic Algorithm, interactions between the initial concentration of Cefixime with reaction time, as well as between the initial concentration of Cefixime and catalyst amount, had the greatest impact on selecting the optimal values. Using the Genetic Algorithm and SOLVER models, the optimum values for the initial concentration of Cefixime, pH, time, and catalyst amount were determined to be (6.14 mg L^−1^, 3.13, 117.65 min, and 0.19 g L^−1^) and (5 mg L^−1^, 3, 120 min, and 0.19 g L^−1^), respectively. Given the presented results, this research can contribute significantly to advancements in intelligent decision-making and optimization of the pollutant removal processes from the environment.

## Introduction

One of the serious issues in the environmental domain is the presence of antibiotics in water sources. The presence of these pharmaceutical substances can have irreplaceable effects on health^1,2^. Negative impacts resulting from the presence of drugs in the environment include the development of drug resistance in humans and animals and leading to a loss of antibiotic effectiveness in disease treatment^3–5^. Furthermore, there are harms to beneficial microorganisms in aquatic and soil environments, adverse effects on animals and plants, a reduction in soil microbial diversity, and the destruction of agricultural productivity and soil structure^6^. Antibiotics can enter the environment through various avenues, with major contributors being the pharmaceutical industry, improper use in agriculture and livestock, incorrect disposal through municipal waste, and excretion from the human body^7,8^.

Controlling the entry of antibiotics into the environment requires collaboration from individuals in industrial, governmental, and environmental sectors, along with proper management measures in this regard. The use of innovative technologies in reducing antibiotics can significantly contribute to improving the environmental situation^9–11^. To delve further into this issue, this study investigates the reduction of the antibiotic cefixime (due to its widespread use in treating bacterial infections and its complex structure) from the aquatic environments using the WO_3_/Co-ZIF photocatalysis process.

Cefixime, with the chemical formula C_10_H_11_N_3_O_3_S, belongs to the Cephalosporin class of antibiotics and exhibits antibacterial effects against both gram-positive and gram-negative bacteria^12–14^. This antibiotic can have a substantial impact on the treatment of skin, gastrointestinal, respiratory, urinary, prostate, and bronchial infections. Moreover, it can pose challenges in ecological settings due to its significant environmental stability^15,16^. Removing this antibiotic from the environment may lead to the easier degradation of similar compounds due to its complex and stable structure. The presence of organic compounds in the structure of this antibiotic is another issue that should not be overlooked. The combination of these organic substances with chlorine in the water treatment systems can introduce carcinogenic and highly hazardous compounds into the environment^17,18^. Therefore, investigating the behavioral trend of this antibiotic in the environment appears to be highly essential.

In recent years, researchers have been seeking efficient methods and new technologies in the field of nanotechnology to achieve high efficiencies in the degradation of complex compounds from the environment^19,20^. The reason for using these nanostructures is their high efficiency and the generation of free radicals with high oxidative potential^21^. These radicals are non-selective and have a high reactivity with antibiotic molecules^22–24^.

In this study, WO_3_/Co-ZIF nanostructure, which is part of Co-based zeolite imidazolate frameworks (ZIF), was used along with tungsten oxide. This nanostructure has a large surface area, good thermal stability, and low toxicity^25–28^. Another advantage of these nanostructures is their easy recyclability for reuse in treatment of the industrial wastewater. In this nanostructure, Co-ZIF and WO_3_ are integrated into a composite form, which increases the stability and resistance against free radicals. Also, this nanocomposite had a synergistic effect to increase cefixime degradation. Additionally, the presence of cobalt with strong catalytic properties in oxidation reactions and the strong structural strength of WO_3_ can make this nanocomposite an ideal platform for environmental purposes in industrial applications^27,29^.

One of the advanced tools in photocatalytic processes and other chemical processes is the utilization of artificial intelligence models. Each machine learning model has its own strengths in data interpretation. For example, ANN is useful in understanding complex nonlinear relationships^30^, while SVR is effective at high scales with less computational cost. By comparing these models, we can identify the most important factors in the removal of cefixime and better understand the mechanisms of photocatalysis. It is important to ensure that the selected machine learning model performs well in real-world conditions, so we evaluate the performance of different models using metrics such as mean absolute error (MAE), absolute root mean square error (RMSE), and R^2^ score^31^. This helps us identify the optimal conditions for cefixime removal. We also use optimization algorithms such as GA and SOLVER to find the best combination of parameters for achieving the highest removal efficiency of cefixime^32^. The optimized conditions identified through this research can be directly applied in real-world settings, aiding environmental remediation efforts and solving water pollution problems caused by pharmaceutical pollutants^33,34^. By accurately predicting the optimal conditions, we can reduce the consumption of catalysts, energy, and time required for the photocatalytic process. This promotes sustainable practices in wastewater treatment and reduces operating costs. Overall, this research not only improves our understanding of photocatalysis processes, but also provides practical solutions for environmentally friendly pollutant removal. It bridges scientific research and real-world applications in environmental remediation^35,36^. The method presented in this research is scalable for industrial applications and is a reliable and repeatable method for large-scale removal of cefixime. Machine learning models, including Artificial Neural Networks (ANN) and Support Vector Regression (SVR), along with Response Surface Methodology (RSM), were employed to enhance the photocatalysis process for cefixime removal from aqueous environments. These models enable a deeper understanding of the underlying mechanisms governing the photocatalytic degradation of cefixime, identify influential parameters, and optimize process conditions to maximize efficiency. The integration of ML models, such as ANN and SVR, with RSM offers a comprehensive approach to optimizing the photocatalysis process for cefixime removal. By analyzing experimental data and identifying complex patterns, these models provide precise predictions of experimental outcomes and facilitate the identification of optimal operating conditions to achieve the best performance in pollutant removal from aqueous environments. These models can determine the best parameters and optimal conditions for each parameter by analyzing advanced experiments and accurately analyzing experimental data^37–39^. This phenomenon helps identify the best-performing materials with high efficiency in pollutant removal systems. These processes can evaluate the synergy and interaction of parameters by analyzing patterns and identifying the exact parameters, providing a better understanding of the processes with high accuracy. Another advantage of artificial intelligence models is the rapid achievement of results in a very short period, which is crucial in reducing costs and research time^40,41^. These models randomly select experimental data for analysis and testing and ultimately evaluate the accuracy of the work^42^. They can also identify and eliminate outlier data that disrupt the system's performance and provide very accurate predictions of results. For this purpose, in this research, two artificial intelligence software (ANN and SVR) and also RSM, were used for better prediction of experimental results, examining outlier data, testing errors, and selecting the most important influential parameter in the photocatalytic process^43^. Additionally, the GA and SOLVER models were used to select optimum values and assessment of the synergistic and interaction effects of the data.

This study introduces a novel approach to tackle antibiotic pollution in water, focusing on cefixime removal via WO_3_/Co-ZIF photocatalysis. It innovates in utilizing advanced nanotechnology, particularly a cobalt nanostructure combined with tungsten oxide, offering high efficiency in pollutant degradation. Additionally, it integrates artificial intelligence models like ANN, SVR, and RSM for precise prediction and optimization of experimental outcomes, enhancing the understanding and efficiency of the photocatalytic process. The study also employs optimization models like GA and SOLVER, ensuring optimal process conditions and assessing synergistic effects among parameters, offering potential solutions for antibiotic pollution in water sources.

## Material and methods

### Preparation of WO_3_ nanoplate and WO3/Co-ZIF nanocomposite

To fabricate WO_3_ nanoplate, the method presented by Zhang et al.^44^ was employed. Initially, 1.0 g of sodium tungstate dihydrate was mixed with 50.0 mL of deionized water under sonication conditions for 15 min. Subsequently, 8.0 mL of lactic acid was added to the previous mixture, and the reaction continued for 30 min. HCl (6.0 M) was used to adjust the pH to 1.0. The resulting mixture was subjected to a temperature of 120 °C for 12 h. The yellowish compound obtained was then washed with deionized water and underwent calcination to generate WO_3_ nanoplate at a temperature of 400 °C for 4 h.

According to our previous studies^27,28^, WO_3_/Co-ZIF nanocomposite was prepared with sol–gel method. 1.2 g of cobalt nitrate hexahydrate was mixed with 3.0 g of the previously prepared WO_3_ nanoplate. This mixture was sonicated in a 50 mL methanol solution for 30 min. To generate microcrystals of Co-ZIF, 3.12 gr of 2-methylimidazole was added to the mixture, and then reaction continued for 24 h. Finally, the purple solution obtained was washed multiple times with methanol to produce WO_3_/Co-ZIF nanocomposite.

### Investigation of catalyst properties

To analyze the structure and properties of the catalyst, two analytical techniques were employed: Atomic Force Microscopy (AFM) by Dual Scope TMDS 95–200/50 apparatus and Scanning Electron Microscopy (SEM) by Sigma VP ZEISS. The AFM technique, operating based on the effects of atomic forces on the sample surface, was utilized for measuring the mechanical properties of the surface and imaging different surfaces of the catalyst. SEM imaging, which utilizes electron beam scattering, was employed for visualizing the surface and internal structure of the catalyst on the nano and micro scales^27,29^.

### Photocatalytic reactor and experimental procedures

The photocatalytic experiments were conducted discontinuously inside a polymethyl methacrylate reactor equipped with a 6 W UVC lamp manufactured by Philips. The UV lamp was kept on for half an hour before the experiments to ensure the sufficient photon intensity required for the reaction. To prevent heat interference during the experiments, a circulating water flow system was employed. The UV lamp was positioned vertically inside the reactor, maintaining a 1 cm distance between the lamp and the reactor walls. The reactor was placed on a mixer, and a magnet was used for stirring the reactor contents. After mixing for a specified period, samples were taken from the reaction vessel and analyzed after centrifugation at 1000 rpm. The remaining concentration of Cefixime was measured using a UV–Vis spectrophotometer with λ_max_ = 286 nm. The percentage of Cefixime removal was calculated according to as Eq. (1).

1 $$\% {\text{ Removal}}\,\,{\text{of }}\,\,{\text{Cefixime = }}\frac{{{\text{Initial }}\,\,{\text{Cefixime}}\,\,{\text{concentration}} - {\text{final }}\,\,{\text{Cefixime}}\,\,{\text{concentration}}}}{{{\text{Initial}}\,\,{\text{Cefixime}}\,\,{\text{concentration}}}}$$

### Experimental design based on the RSM model in R software

All experiments were conducted based on the designs generated by the Response Surface Methodology (RSM) model in the R software. To achieve this, the "library (rsm)" was defined and installed in the R software. The considered parameters for designing the runs included initial Cefixime concentration in the range of 5–20 mg L^−1^ (Eq. (2)), pH in the range of 3 to 9 (Eq. (3)), time in the range of 5–120 min (Eq. (4)) and catalyst dosage in the range of 0.5–1.9 gr L^−1^ (Eq. (5)). After defining the variable ranges, coding formulas for the existing ranges were presented in Eqs. (2–5).

2 $$x_{1} \sim \, \left( {{\text{Initial}}\,\,{\text{Cefixime}}\,\,{\text{concentration}}\,\, - \,\,12.5} \right)/7.5$$

3 $$x_{2} \sim\left( {pH - 6} \right)/3$$

4 $$x_{3} \,\sim\,\left( {{\text{Time}}\, - \,62.5} \right)/57.5$$

5 $$x_{4} \,\sim \, \,(WO_{3} {\text{/Co}} - {\text{ZIF}}\,\,{\text{dosage}} - \,0.12)/0.07$$

Subsequently, a Central Composite Design (CCD) was generated, considering 8 axial points and 7 center points (unify. prec.). After editing the data, 39 experimental runs were designed. After designing the runs, the response for each run was determined. After obtaining the response for each run, the coding was performed corresponding to the target table. Based on the obtained codes, fitting the experimental data was carried out using three RSM models: First Order Model, First Model with Interaction, and Second Order Model^45,46^. Further evaluation included assessing the Lack of Fit for various models. The model with a non-significant Lack of Fit was considered the best model. If none of the models had a significant Lack of Fit, the reduced model was employed, removing outliers and data points with high residual values. After selecting the appropriate model, regression results related to the coded model were calculated, and the corresponding formula was presented. Predictions were then made based on the selected model. Finally, contour and perspective plots were generated for the different interaction scenarios in the regression model's output. Based on the best model selected and different modes of interactions in the model regression output, contour, and perspective diagrams were drawn.

### Artificial neural network (ANN) model

The designed runs in the R software and the experimental responses obtained in the laboratory were utilized in this model. Python 3.12 was employed to set up and run this model. Relevant Python libraries for the ANN model were initially installed. The dataset was loaded from an Excel file. Assuming the dataset columns were included X_1_, X_2_, X_3_, and X_4_, and the response is removal (%), the X and Y values were determined. For the X variable assignment, all rows and columns except the last one was selected. For the y variable, all rows and only the last column were chosen (i.e., the dependent variable was selected from the last column of the data). Therefore, in this step, inputs (X) and outputs (y) were separated in the neural network model. Following this, standardization or normalization of features was performed, where the input features were transformed to a consistent scale or standard scale. Standardizing the features helps the model perform better and more stably under the different input feature conditions. Then, the data were randomly split into training and testing groups, defining X-train, X-test, Y-train, and Y-test items. The construction of the neural network model was carried out. For the design of the neural network, the table generated in the R software was utilized in Table S1 of the Supporting Information (SI). After loading the data into Python software and normalizing it, a neural network with 4 input layers, 4 hidden layers, and 1 output layer was initialized. An ANN model with several linear layers (`Linear`) and ReLU activation functions was designed. Subsequently, 20% of the data was assigned for testing and 80% for training. This neural network layer consisted of four hidden layers, representing a Deep Neural Networks design. The Adam optimization algorithm was utilized for optimizing model parameters (including weights and biases). The learning rate for updating the weights was determined to be approximately 0.1. Twenty percent of the data was used for testing, and 80 percent for training. Prediction was performed on the test and training datasets. Evaluation of the test and training datasets was done using evaluation metrics, including MAE (Mean Absolute Error), RMSE (Root Mean Squared Error), and R^2^ Score^47^.

### Support vector regression (SVR) model

Similar to the ANN model, the SVR model was designed using data generated by the R software. After reading the data using the software, the data was stored in Pandas (`data`). Subsequently, preprocessing was performed on the input data (X) and response variable (Y). After scaling the data with a specific scale, random samples were separated into test and training sets. The model was then constructed and trained. Similar to the ANN model, the SVR model was employed to predict removal (%) values. Anaconda and Jupyter environments were used for data analysis. Initially, the desired data were loaded, and preprocessing steps were performed to extract input features (X) and output responses (Y) from the data. The data were then normalized to facilitate the training process and improve predictions for new data. Normalizing the data enhanced the model's performance and accuracy. Subsequently, the data were randomly divided into training and testing groups, defining X-train, X-test, Y-train, and Y-test items. The percentage of data used for testing and training was 20% and 80%, respectively (these data were randomly selected). Next, an SVR model was created using the Linear SVR class and the best-specified parameters. The model was trained using the training data. Predictions were made on the test and training datasets. The error metrics, including MAE, RMSE, and R^2^ Score, were calculated to evaluate the model's performance. Finally, the feature importance in the model was determined using Average Weight Magnitude^43^.

### Optimization models: GA and SOLVER

To exploit the optimization models with GA artificial intelligence software and SOLVER software, it is necessary to provide non-coded regression coefficients by taking lm from the coded regression results in R software. The SOLVER software can also be utilized as an optimization tool to find optimal values for parameters. This software employs various optimization algorithms to determine the best parameter values for the objective function. In the SOLVER model, after inputting the regression coefficients from R, the desired formula was written in the SOLVER software. The upper and lower bounds for each parameter were set, the object was specified, and the SOLVER software was instructed to handle changeable cells. Constraints for each variable were defined^48^. By executing the command in the SOLVER software, and considering the necessary limitations, it was provided the best optimization results. Utilizing GA offers significant advantages due to its randomized mechanisms, particularly effective in solving problems with a large number of variables. The software can obtain optimal local or global points by enhancing the population through evolutionary processes across generations. The interactive population features make it highly beneficial for practical and industrial optimization scenarios. GA optimization was done in Anaconda software and the Jupiter environment. Initially, the required libraries for the GA model were installed. The adjusted size value for the system was determined, and the objective function for the model was defined. Necessary adjustments were made to maximize the objective function. Genetic algorithm settings included the number of individuals, generations, and mutation rate. The minimum and maximum ranges for each data were specified and entered into the software. Next, the initial population was created with the new ranges. Arrays were initialized to store the best and mean fitness values in each generation. The parameters were restricted to valid ranges, and fitness values were calculated for each individual in the population. The best and mean values were added to the appropriate arrays. Parents were selected based on the probability of backtracking. Offspring were generated using crossover and mutation. Offspring replaced parents in the population. Interaction plots for all pairs were generated. A grid of X values for the current pair was created, and fitness values were calculated for each combination of X and Y. The interaction plot was then drawn. Layout adjustments were made to prevent clipping of titles and vertical space between subplots was increased. Also, the convergence curve with the best and mean fitness values was plotted. In the end, the best solution and optimal removal for the current model were presented ^49,50^.

## Results and discussion

### Catalyst characteristics

**Figure 1:**
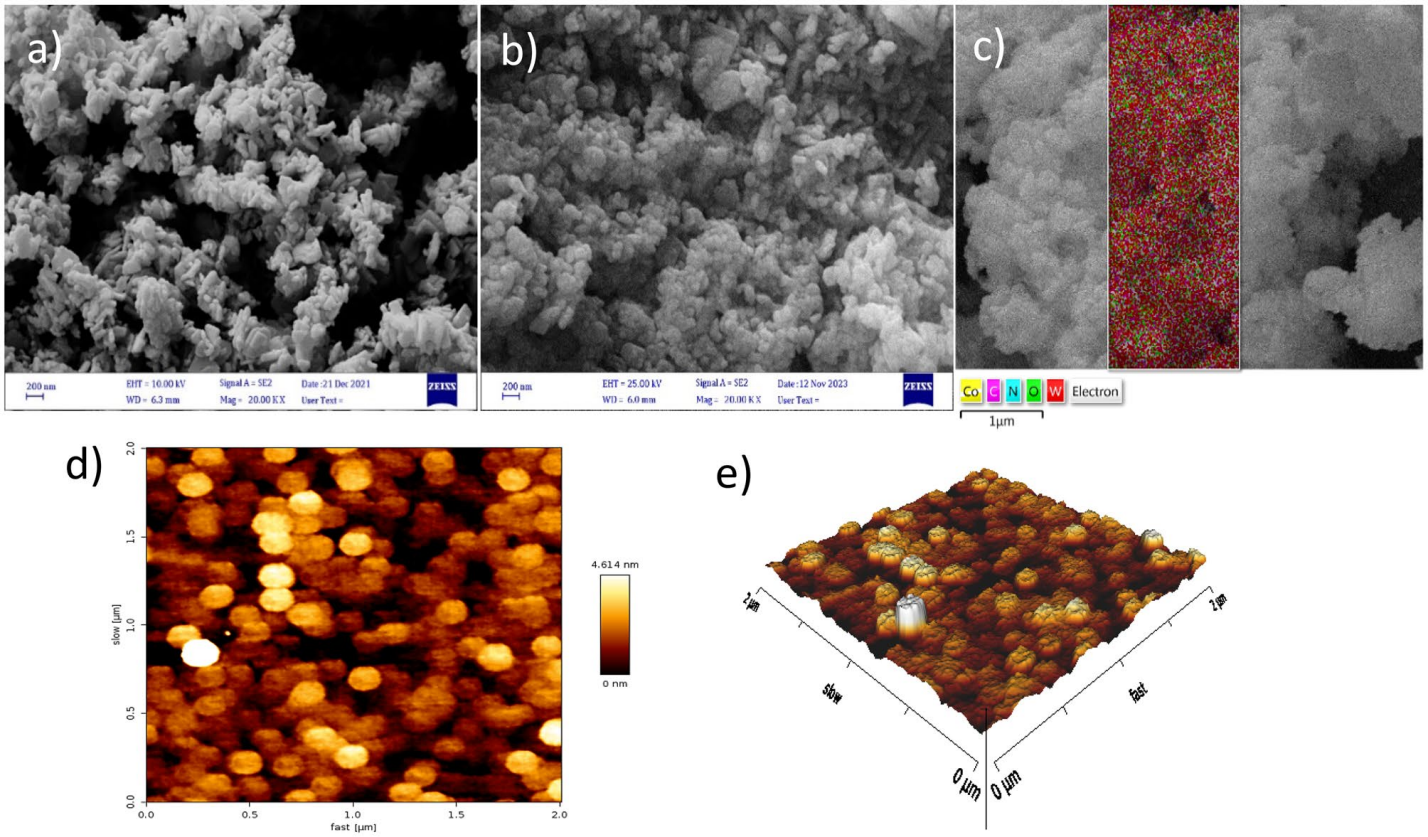
SEM images of WO_3_ (**a**), WO_3_/Co-ZIF (**b**) and SEM mapping of WO_3_/Co-ZIF (**c**); 2D and 3D AFM analysis of WO_3_/Co-ZIF (**d**, **e**).

SEM images were employed to investigate the structure and morphology of WO_3_ and WO_3_/Co-ZIF nanocomposite. The presence of a layered structure in WO_3_ with a thickness of approximately 90 nm (Fig. 1a) is clearly evident. Following the deposition of Co-ZIF crystals on WO_3_, no significant alteration in the initial structure of the two compounds was observed (Fig. 1b). Moreover, SEM mapping confirmed the homogeneous structure of the nanocomposite, verifying the presence of Co, C, N, O, and W elements in the nanocomposite structure (Fig. 1c). Moreover, AFM analysis provided insights into the surface topology of the WO_3_/Co-ZIF nanocomposite (Fig. 1d), corroborating the results presented in the SEM analysis regarding surface morphology and the homogeneous structure of the nanocomposite. This analysis of pores was conducted in the 2.0 µm × 2.0 µm region (Fig. 1e). In the 2D and 3D images presented in bright regions, a better understanding of the sample's surface structure was achieved. The RSM roughness was determined to be approximately 1.049 nm. Additionally, the length of the line was determined to be around 516.5 nm. Furthermore, a nominal diameter of 138.3 nm was assigned to this nanocomposite.

### Design based on RSM model

The objective of designing based on this model is to aid in analyzing the simultaneous effects of the various variables on a response. After the design matrix was generated using CCD in the R software, the data and responses were fitted with three RSM models (Factorial model, Quadratic model and Factorial-quadratic model). These three models were utilized for statistical analysis and designing response surface models, these models are included in the supplementary file. A comparison of the three RSM models with the mentioned criteria is presented in Table 1.

**Table 1:** The final results obtained from the comparison of three models (coded date).

Model	F-statistic (p-value)	R-squared (R^2^)	AIC	RSS	Lack of fit
Factorial model	56.15 on 4 and 34 DF (p-value: 1.653e−14)	0.8685	159.33	1794.7	7.965e−06
Quadratic model	35.21 on 10 and 28 DF (p-value: 3.194e−13)	0.9263	148.74	1005.6	6.304e−05
Quadratic factorial model	249.4 on 9 and 29 DF, p-value: < 2.2e−16	0.9872	78.35	174.1	0.6635

Based on the results presented in Table 1, it is evident that the Quadratic Factorial Model, with higher values of F-statistic, R-squared (R^2^), and lower AIC, RSS, and p-value compared to the other two models, as well as having an insignificant Lack of Fit, can be considered as the best model for aligning with the data. Therefore, the Quadratic Factorial Model was utilized for predicting results and designing the relevant formula. The ANOVA analysis for the Quadratic Factorial Model was presented in the following Table 2 (x_1_ = initial Cefixime concentration (mg L^-1^), x_2_ = pH, x_3_ = Time (min), x_4_ = catalyst dosage (g L^−1^)).

**Table 2:** Analysis of variance (ANOVA) for Quadratic Factorial Model (coded date).

Parameter	DF	Sum of squares	Mean square	F-value	Probability (P)
FO (x_1_, x_2_, x_3_, x_4_)	4	11,855.2	2963.81	493.6005	< 2.2e−16
PQ (x_2_, x_3_)	2	839.7	419.83	69.9199	8.061e−12
TWI (x_2_, x_3_, x_4_)	3	780.9	260.31	43.3522	7.687e−11
Residuals	29	174.1	6.00		
Lack of fit	15	80.4	5.36	0.8007	0.6635
Pure error	14	93.7	6.69		

In the ANOVA table, it is entirely evident that in the Quadratic Factorial Model, four independent variables (FO (x_1_, x_2_, x_3_, x_4_)), the second power of variables (PQ (x_2_, x_3_)), and the interactions between variables (TWI (x_2_, x_3_, x_4_)) are significant and play a fundamental role in the model's alignment with the corresponding data. The significant p-values in the first three rows of the ANOVA table emphasize this fact. Therefore, based on the results presented in the ANOVA table, the regression table related to the Quadratic Factorial Model was designed and provided in Table 3. It is worth noting that all the designs for RSM in the R software are based on the encoded data. Based on Table 3, the corresponding formula for predicting the results was provided in Eq. (6). The predicted results of the model were presented in Table S1 of the supporting information (SI).

6 $$\begin{aligned} {\upgamma }\,{ = }\, & {34}{\text{.008}} - {4}{\text{.54X}}_{{1}} - {38}{\text{.94X}}_{{2}} { + 16}{\text{.25X}}_{{3}} { + 13}{\text{.20X}}_{{4}} { + 18}{\text{.23X}}_{{2}}^{{2}} \\ & { + 7}{\text{.83X}}_{{3}}^{{2}} - {17}{\text{.22X}}_{2} {\text{X}}_{3} - {13}{\text{.17X}}_{2} {\text{X}}_{4} + 17.62{\text{X}}_{3} {\text{X}}_{4} \\ \end{aligned}$$

**Table 3:** Regression analysis for Quadratic Factorial Model (coded date).

Model term	Coefficient estimate	Std. error	t-Value	*p*-Value
Intercept	34.00833	0.53472	63.6001	< 2.2e−16
x_1_	− 4.54167	1.00037	− 4.5400	9.091e−05
x_2_	− 38.94167	1.00037	− 8.9272	< 2.2e−16
x_3_	16.25833	1.00037	16.2523	4.172e−16
x_4_	13.20833	1.00037	13.2034	8.589e−14
x_2_^2^	18.23125	1.70147	10.7150	1.347e−11
x_3_^2^	7.83125	1.70147	4.6026	7.644e−05
x_2_:x_3_	− 17.22500	2.45040	− 7.0295	9.897e−08
x_2_:x_4_	− 13.17500	2.45040	− 5.3767	8.907e−06
x_3_:x_4_	17.62500	2.45040	7.1927	6.434e−08

### Influence of interaction between variables on the dependent variable (response) using contour and perspective plots

Based on ANOVA analysis, the impact of interaction between independent variables on the response (dependent variable) was investigated by designing Contour and Perspective plots. Contour and Perspective plots for examining the interaction effects between x_2_:x_3_, x_2_:x_4_, and x_3_:x_4_ were presented in Fig. S2, SI. In Figs. S2 (a) and S2 (d), the interaction effect between the independent parameters x_2_ (pH) and x_3_ (Time) were examined. As evident, when the value of x_2_ is 4.5, increasing the value of x_3_ enhances the performance, especially when it is noticeable at values lower than 4.5. In x_2_ values above 4.5, with the simultaneous increase of two parameters, no change in removal performance was observed. The reduction in the x_2_ variable simultaneously with an increase in the x_3_ variable leads to an improvement in the performance of the photocatalytic process. This could have various reasons, one of which might be the alteration in the photocatalyst structure with changing pH. It is plausible that at lower x_2_ levels, the size and shape of the nanoparticles change, improving light adsorption and photocatalytic activity. Additionally, at lower pH levels, the number of reactive sites on the surface of the photocatalyst increases, enhancing photocatalytic activity. Increasing doping and photocatalyst oxidation by changing x_2_ could be another reason for the improvement of the photocatalytic activity at the lower x_2_ levels. The decrease in pH facilitates the phase transfer process from liquid to solid (e.g., removal of by-products), enhancing photocatalytic activity. It is also possible that the catalyst conditions at lower x_2_ change increase selectivity by radicals present in the reaction. A decrease in the pH, due to the increased interaction of light with active materials at the reaction site and also the generation of electron–hole pairs, can improve the photocatalytic activity^51^. Furthermore, the decrease in pH can enhance the optical properties (light adsorption) of the catalyst, leading to increased efficiency. In Figs. S2 (b) and S2 (e), the interaction effect of x_2_ (pH) and x_4_ (catalyst amount) on the removal performance of Cefixime was examined. It is observed that with a decrease in x_2_ and an increase in x_4_, the efficiency removal of Cefixime increases. Moreover, an increase in the catalyst amount in the reaction environment facilitates electron transfer^52^. Additionally, reducing the activation energy with an increase in the catalyst amount can accelerate the photocatalytic reaction rate. In some cases, catalysts may degrade in the reaction environment; increasing the catalyst amount improves its resistance to erosion. However, it should be noted in pH values above 4.5, an increase in the catalyst amount did not affect the removal performance. Based on Figs. S2 (c) and S2 (f), it was observed that simultaneous increases in x_3_ and x_4_ lead to an increase in photocatalyst efficiency. The plots clearly show that below 80 min of reaction time, increasing the catalyst amount has no effect on removal efficiency, and above 80 min of reaction time, increasing the catalyst amount (x_4_) significantly enhances removal efficiency. Therefore, according to Fig. S4, acidic pH values, a time above 80 min, and a catalyst amount above 14.0 g L^-1^ demonstrate the best performance in photocatalytic removal efficiency ^27,37^.

### Neural network (ANN) model

**Figure 2:**
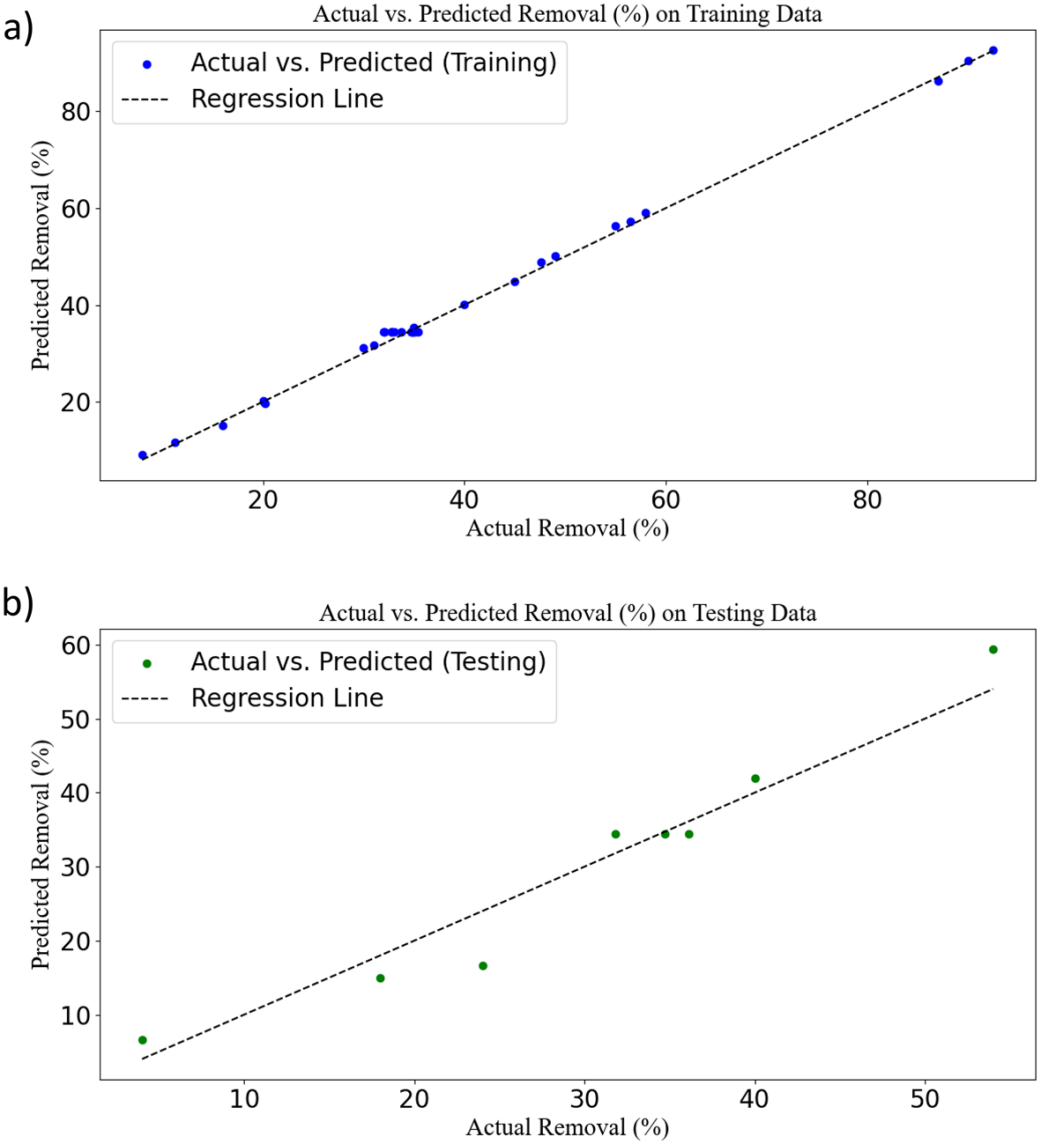
Scatter plot of predicted values from the ANN versus actual values for training (**a**) and testing of data (**b**).

After training the data, the predicted results for the test and training data were calculated and presented in Fig. 2. In this figure, predicted vs. actual data was plotted for both test and training datasets. These plots can serve as a crucial tool for evaluating the performance of the ANN model. In these plots, the X and Y axes represent the values of actual and predicted, respectively. Each point on these plots represents a sample from the test and training data. The closeness of the data to the regression line or the location of the data on the regression line indicates the high accuracy of the model in data analysis. In the results related to the test data (8 tests out of 39 tests were included and were randomly selected), a slight deviation from the regression line was observed, while this deviation was much less in the 31 data related to training, and most of the points were on the regression line^38^.

### Evaluation of residuals for training and testing data for ANN model

**Figure 3:**
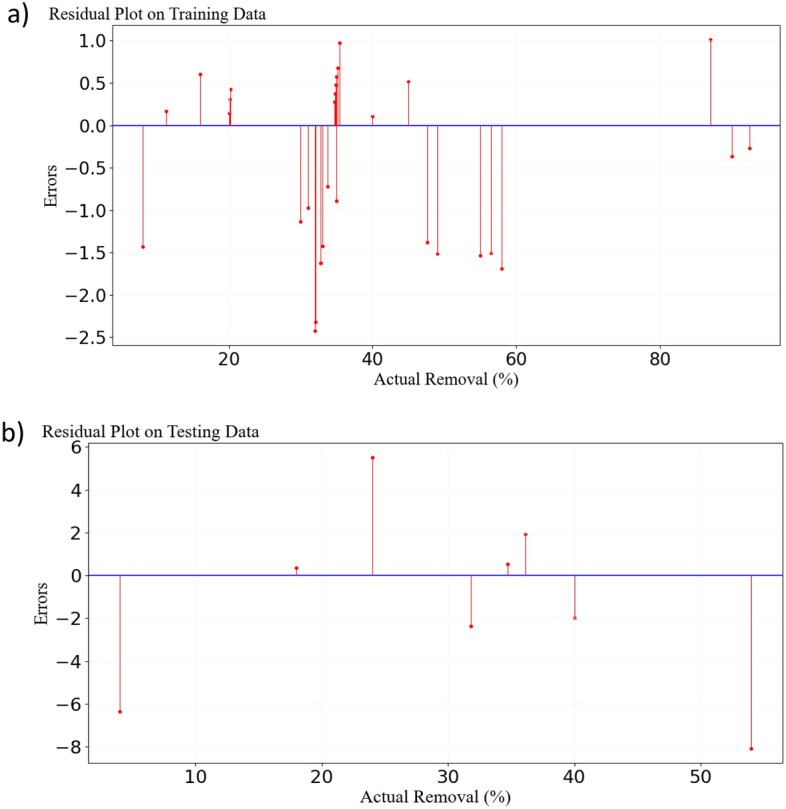
Scatter plot of residual values from the ANN for training (**a**) and testing of data (**b**).

To further investigate, residual values were computed for both test and training datasets, and the corresponding plots were presented in Fig. 3. In other words, the difference between the actual (observed) and predicted values of the model was calculated, and the results are visualized in Fig. 3. Indeed, these plots were utilized to examine the distribution of errors and the prediction accuracy of the model under scrutiny. In a good model, residuals should be randomly scattered and close to zero. This situation indicates a good prediction of the model on both test and training data. A plot with a specific and non-random pattern indicates that there are structures or patterns in the data that the model failed to capture. In such cases, there is a need to improve the model or modify input features. The presence of significant differences between residuals indicates the existence of outliers that need to be removed from the data. In the present study, due to some large differences in the residuals, it was necessary to remove outliers. A good residual plot includes scattered points around the zero line, and the points are randomly and uniformly distributed. By examining the plots in Fig. 3, it is observed that in both the test and training residual plots, the data points are well scattered around the zero line. Upon closer inspection, it is evident that the data points were chosen completely randomly. Additionally, the homogeneity of the data points to each other is visible in both plots. Furthermore, to examine the model's performance more thoroughly, additional evaluation metrics such as MAE (Mean Absolute Error), RMSE (Root Mean Squared Error), and R^2^ Score were scrutinized for both test and training datasets. The evaluation results of the model indicated that the values for MAE, RMSE, and R^2^ Score for the testing data were 3.22, 3.91, and 0.92, respectively; while for the training data, these values were 0.9, 1.02, and 0.99, respectively^40,53^. The higher values of MAE and RMSE, along with the lower R^2^ Score for the testing data compared to the training data, are visible in Fig. 3.

### Model performance evaluation and feature importance for the ANN model

**Figure 4:**
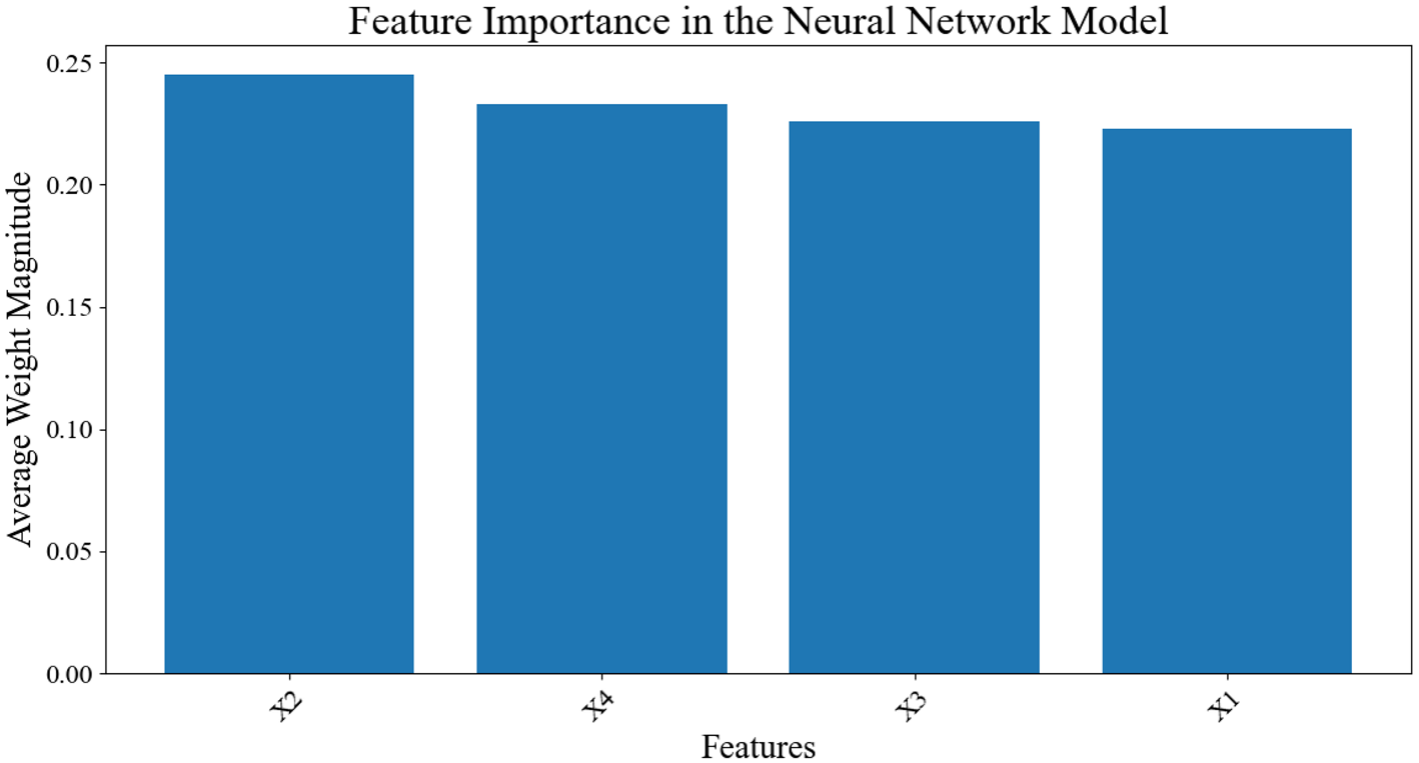
The feature importance in the ANN model.

To investigate the importance of features in the ANN model, the feature importance in the neural network model plot was employed (Fig. 4). This plot illustrates the importance of each parameter and its impact on predicting the model results. The plot depicts the importance of parameters with a bar chart, showing the importance of each parameter from largest to smallest. Thus, parameter x_2_ has the highest importance, and x_1_ has the least importance. In other words, parameter x_2_ has the most significant effect on predicting the model results. However, since all the plots are trending upwards (positively), it indicates that an increase in the values of these four parameters has a positive impact on the model's output^38^. The prediction results of the model are presented in Table S1, SI.

### Designing SVR model

**Figure 5:**
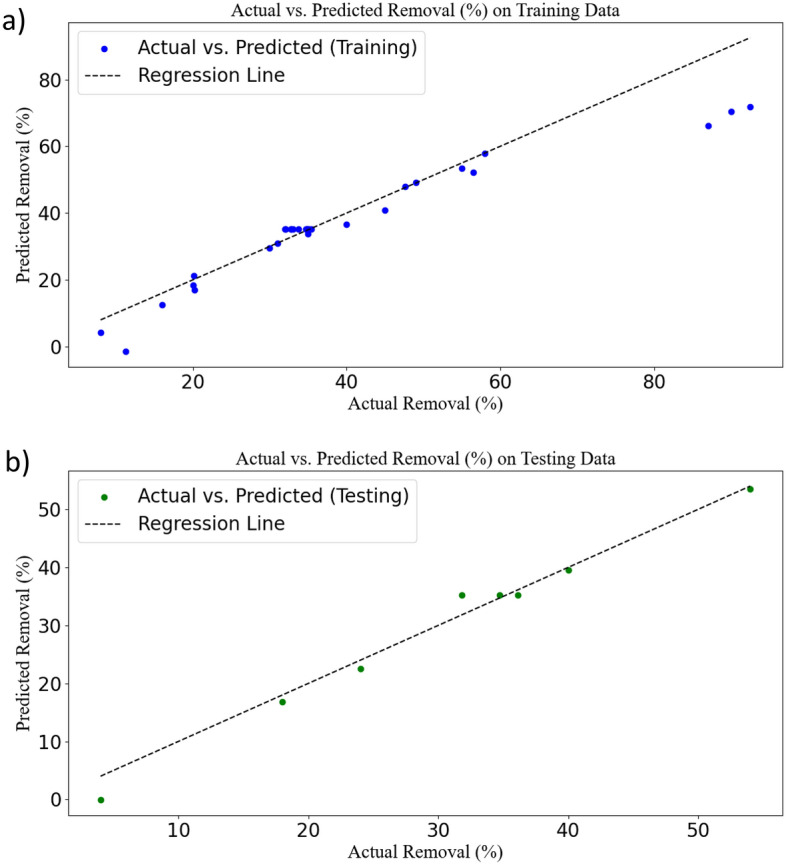
Scatter plot of predicted values from the SVR versus actual values for training (**a**) and testing of data (**b**).

For interpreting the output results from the SVR software, several plots were utilized. Initially, a plot of actual data versus predicted model data for random test and training data was created^54^. The results obtained are presented in Fig. 5. Interpreting these plots provides a better understanding of the SVR model's performance. In contrast to the ANN model, it was observed that in the plot related to test data, the points are very close to the regression line, indicating low error and excellent model performance on training data. Regarding the regression plot related to training data, some of the data points were scattered around the regression line, suggesting potential overfitting, even though a significant number of points lie directly on the regression line^55^.

### Further analysis and evaluation metrics for the SVM model

**Figure 6:**
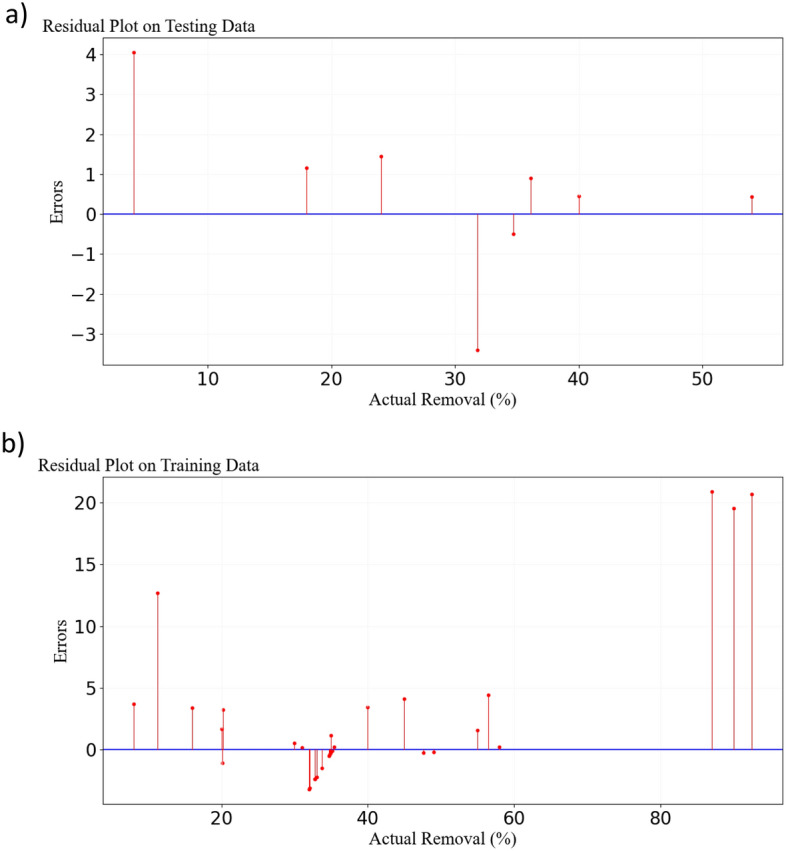
Scatter plot of residual values from the SVR for testing (**a**) and training of data (**b**).

For a more in-depth examination of outliers present around regression lines, residual plots were employed (Fig. 6). It can be observed that there are 2 and 4 outliers or errors in test and training data, respectively. This discrepancy might be associated with the model's failure to adapt to the relevant test and training data, leading to incorrect predictions. Another reason could be the model's dependence on specific features. Also, errors in test data may stem from features that are absent in the training data. The different distribution of test data compared to training data could be another reason for these errors. Outliers can also contribute to significant errors in the model. Furthermore, to analyze the model further, evaluation metrics such as MAE, RMSE, and R^2^ Score were examined. According to the results extracted from the SVM software, the values for MAE, RMSE, and R^2^ Score for test and training data were determined to be (1.54, 2.02, 0.98) and (3.85, 2.02, 0.88), respectively. These results indicate that for test data, the MAE is 1.54, signifying that a lower MAE can be more effective in predicting test data. Additionally, an RMSE of less than 2.03 can indicate high accuracy in predicting test data. The R^2^ Score value of 0.98 indicates that the model's interpretation of test data was well done, and the model has adapted well to the test data. Furthermore, the high R^2^ Score signifies the ability of the model to explain 98% of the changes in the response variable. It's important to note that test data are the most critical parameter for evaluating performance. These data indicate how well the model can adapt to unknown data. The better the test data is, the better the model performs on the new data. On the other hand, it should be noted that the training data is used to train the model, and the basis of the model prediction is based on the conditions of the training data. In conclusion, considering the presented results from the test data, it is evident that the model has a very good performance in predicting results, and the MAE and RMSE values indicate very low errors in the model. Therefore, the displayed error values in the model (Fig. 6) are not likely to significantly interfere with the model's performance in predicting test results^43,55^.

### Model performance evaluation and feature importance for the SVR model

**Figure 7:**
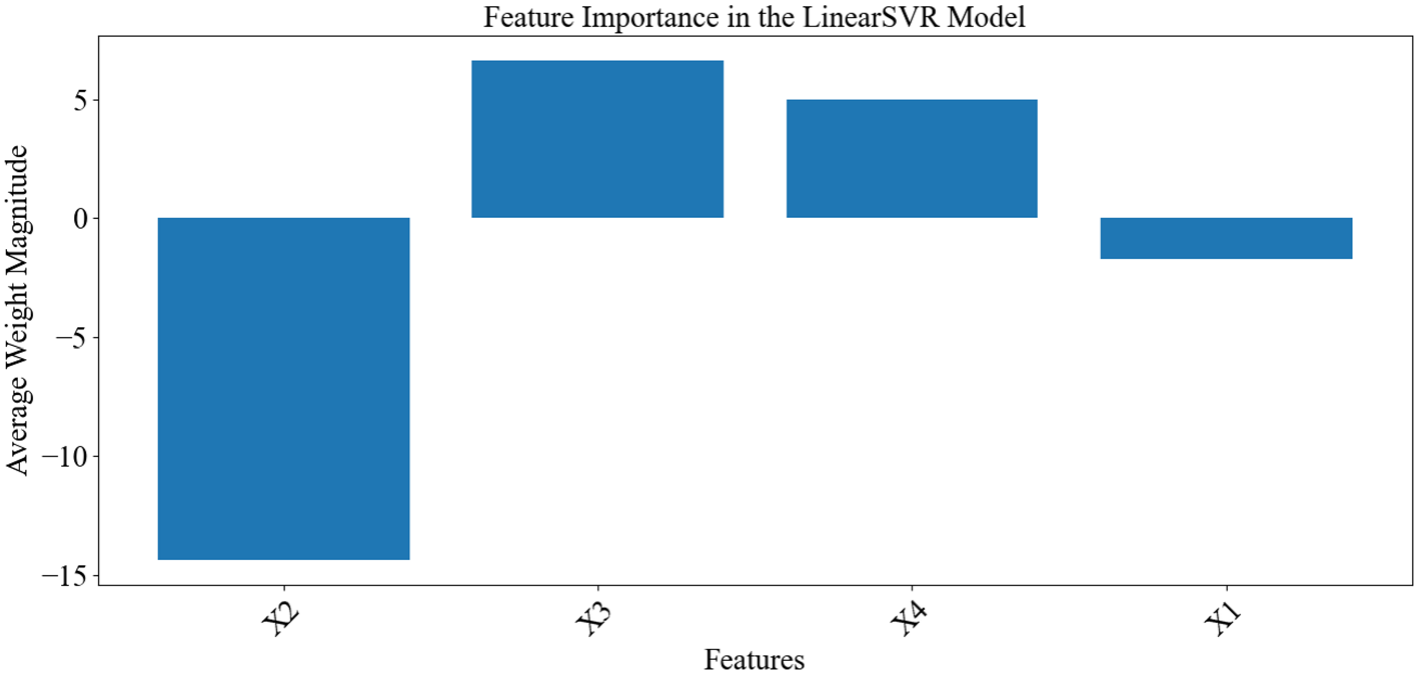
The feature importance in the SVR model.

The importance of each parameter was evaluated based on its weight in the SVR model (Fig. 7). This plot assisted in determining the level of influence of each parameter in the model's decision-making process. According to Fig. 7, Through the presented weights, the impact of each variable in predicting the results can be determined. Based on Fig. 7, three weight groups are evident: a group with very high positive weights, a group with very high negative weights, and a group with weights close to zero. Parameters such as x_3_ and x_4_, which have significantly positive weights, indicate an increase in the positive model output prediction with an increase in these two parameters. Conversely, an increase in the parameter x_2_ leads to a decrease in the positive model output prediction, playing a crucial role in negative model decisions^43^. Parameter x_1_, with a negative weight close to zero, suggests minimal influence on the model's decision-making and indicates that the model pays little attention to this parameter. Consequently, this parameter has a negligible role in the final model prediction. It is important to note that the sign of a parameter, whether positive or negative, is not indicative of its importance in the model's decision-making. Instead, the weight assigned to each parameter determines its significance in the model's decision-making process^55^. Therefore, according to the SVR model, the most and least important parameters in decision-making are x_2_ and x_1_, respectively. The model prediction results are presented in Table S1, SI.

### Comparison between different models in terms of fitness and interpretation of the provided data

To compare the performance of the SVR and ANN models:

A. Data Preparation and Model Design:
  Both models utilized data generated by R software.
  In the ANN model, data was normalized, and a neural network with 4 input layers, 4 hidden layers, and 1 output layer was initialized. The ANN model employed linear layers and ReLU activation functions.
  Similarly, the SVR model employed random sample separation into test and training sets after preprocessing the data in Pandas.
B. Model Performance Evaluation:
  The performance of both models was evaluated using various metrics such as Mean Absolute Error (MAE), Root Mean Squared Error (RMSE), and R^2^ Score.
  For the ANN model, the MAE, RMSE, and R^2^ Score for the testing data were 3.22, 3.91, and 0.92, respectively, and for the training data, these values were 0.9, 1.02, and 0.99, respectively.
  For the SVR model, the MAE, RMSE, and R^2^ Score for the test data were 1.54, 2.02, and 0.98, respectively, and for the training data, these values were 3.85, 2.02, and 0.88, respectively.
C. Residual Analysis:
  Residual plots were utilized to analyze the distribution of errors in both models.
  The ANN model showed good performance with residuals well scattered around the zero line for both test and training data.
  Similarly, the SVR model exhibited good performance with minimal errors, as evidenced by the residuals closely distributed around the zero line for both datasets.
D. Feature Importance:
  Both models employed feature importance analysis to determine the impact of each parameter on the model's decision-making process.
  Parameters with higher weights were considered more influential in both models. In the ANN model, parameter x_2_ was identified as the most important, whil in the SVR model, parameters x_3_ and x_4_ had the highest positive weights.
  Overall, both models demonstrated good performance in predicting the outcomes, with slight variations in their evaluation metrics and feature importance analysis. While the ANN model showed slightly higher accuracy metrics for the testing data, the SVR model exhibited lower errors and better adaptation to the training data. Based on the comparison, it appears that the SVR model outperforms the ANN model. This conclusion is drawn from the lower values of MAE and RMSE, and the higher R^2^ Score for the SVR model compared to the ANN model.

### Optimization with GA and SOLVER models

To optimize the parameters, Artificial Intelligence (AI) software such as Genetic Algorithm (GA) and SOLVER were employed. After defining the objective function for the GA software, crucial parameters, that significantly impact the process, were input into the software^56^. These essential parameters were extracted from non-coded data using Eq. (7), previously prepared in the R software. The formula was derived through regression analysis based on the non-coded parameter values. The regression analysis for the non-coded values of the parameters was provided in Table S2 in the supporting information file. After determining the important parameters through the relevant formula, the range of each variable was specified and input into the software. The goal of this step is to constrain the parameter space for the software. Subsequently, the initial population was defined, and the desired operations were selected. Following that, the processes of crossover, mutation, and population updating, along with the evaluation of the fitness function, were executed. In some cases, it may be necessary to adjust the parameters of the genetic algorithm or the search space; in such cases, the mentioned steps need to be repeated to achieve the optimal result^57^.

7 $$\begin{aligned} {\upgamma }\,{ = }\, & {111}{\text{.5}} - {0}{\text{.6X}}_{{1}} - {23}{\text{.5X}}_{{2}} { + 0}{\text{.06X}}_{{3}} { + 291}{\text{.4X}}_{{4}} { + 2}{\text{.02X}}_{{2}}^{{2}} { + 0}{\text{.002X}}_{{3}}^{{2}} \\ & - {0}{\text{.099X}}_{2} {\text{X}}_{3} - {62}{\text{.7X}}_{2} {\text{X}}_{4} + 4.37{\text{X}}_{3} {\text{X}}_{4} \\ \end{aligned}$$

**Figure 8:**
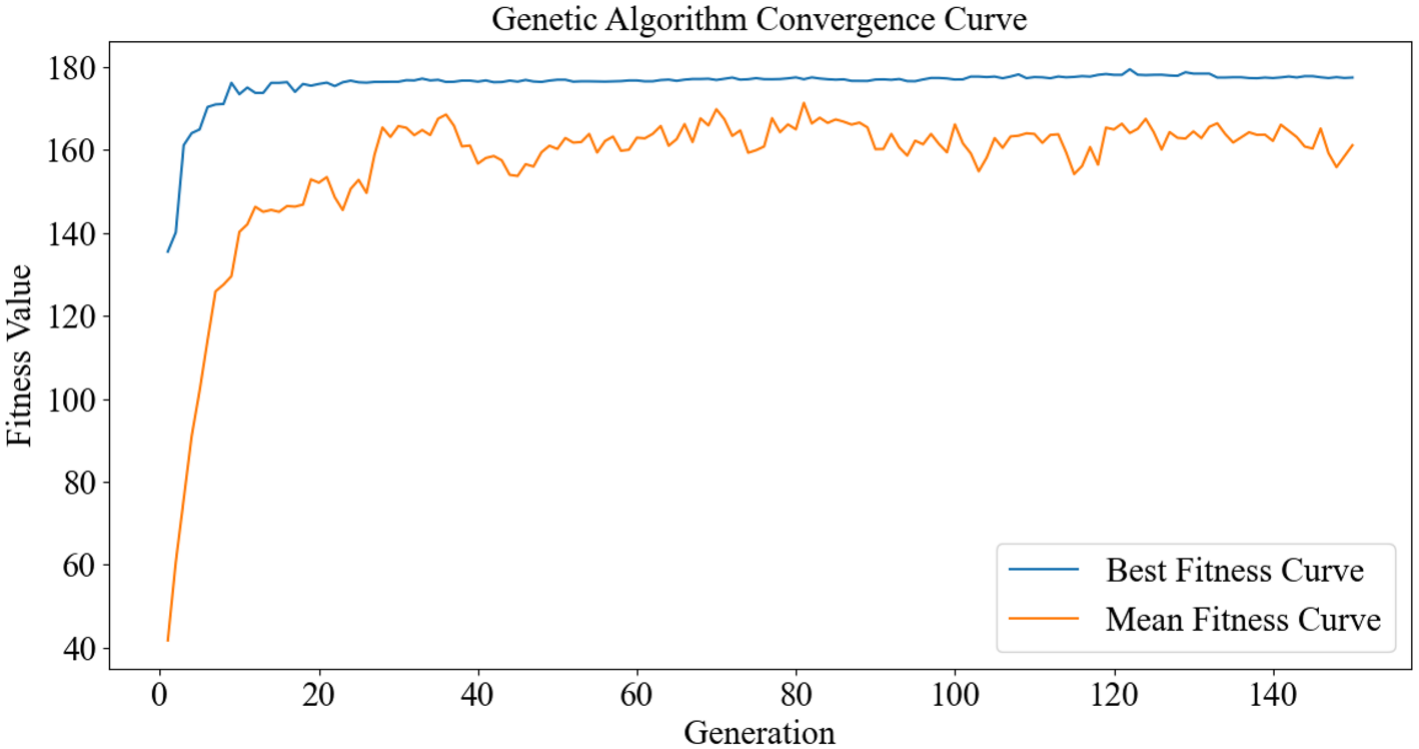
The convergence plot for the genetic algorithm.

The convergence chart for the genetic algorithm is presented in Fig. 8. This chart has two variables, "Generation" and "Fitness Value," which play a crucial role in the progress of the genetic algorithm. It illustrates the changes in the fitness value over generations. In this chart, "Generation," representing the respective population, has been set at 150, and these individuals or populations have evolved from the previous generation. The number 150 indicates the execution of the genetic algorithm up to the last 150th generation, and the results are presented based on this number of generations. The number 180 for the fitness value represents the fitness value in the corresponding generation, indicating the overall improvement in the algorithm's performance at that moment. These results depict the execution trend of the algorithm at a specific stage, showing how optimal each parameter is in the search space and allowing adjustments to each parameter's value in each generation for further optimization. Two values, "Best Fitness Curve" and "Mean Fitness Curve," are observable in the chart. These curves depict two different states of the algorithm. "Best Fitness Curve" shows the best fitness and displays the points indicating the best fitness conditions in the target generation. The higher the points, the better the model has reached better solutions. The "Mean Fitness Curve" chart indicates the algorithm's progress in improving the overall fitness of the population. According to the Mean Fitness Curve chart, it is evident that the curve quickly tends towards the optimal value, indicating rapid convergence. Although oscillatory transitions are observed in the chart, indicating issues such as encountering local slopes in the search space. Therefore, based on the observed charts, it is clear that the algorithm easily reached the optimal values for the parameters.

**Figure 9:**
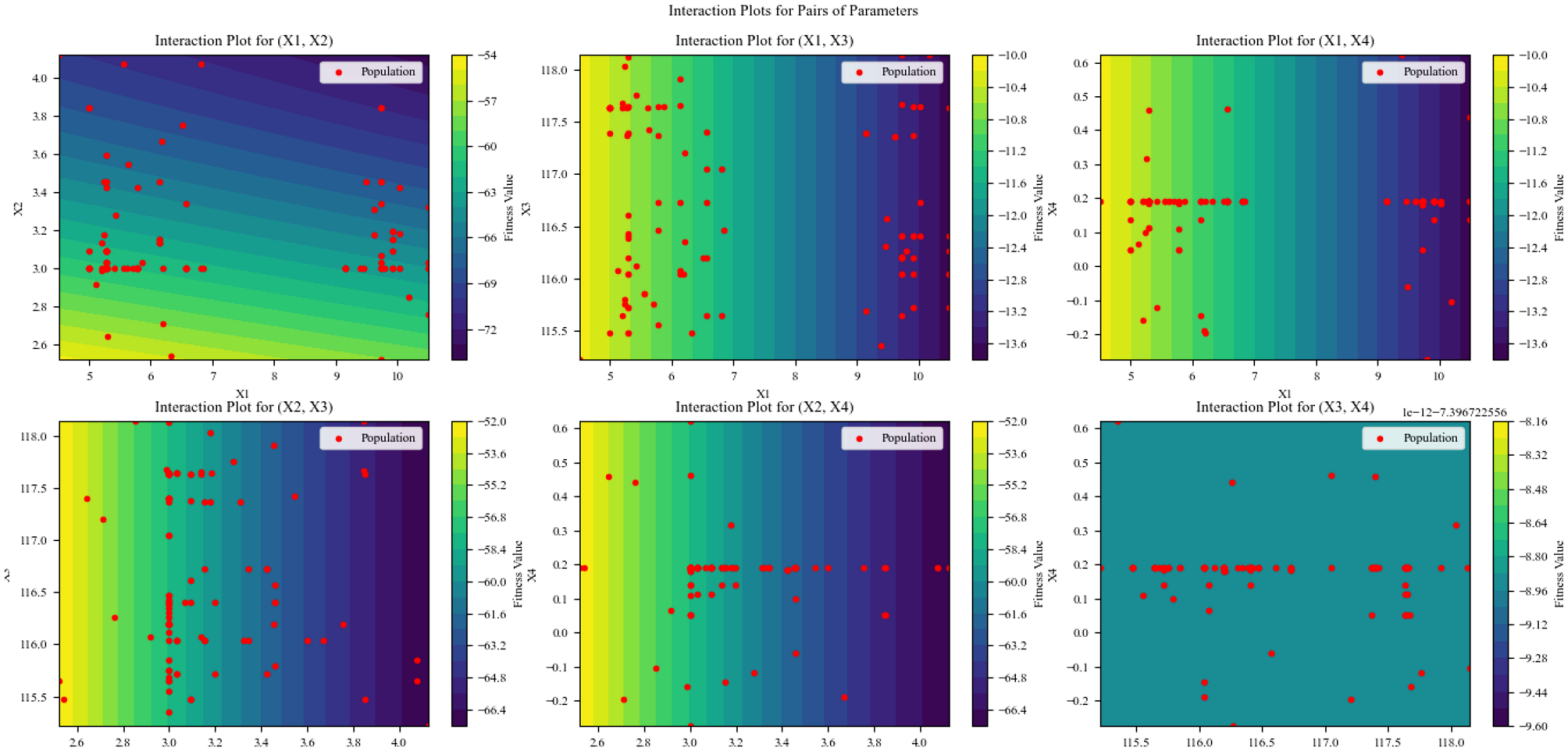
Interaction plots for pairs of parameters.

Interactive charts were designed and plotted for all parameter pairs in the genetic algorithm (Fig. 9). In these charts, the interactive effect between two parameters in a genetic algorithm was examined and analyzed. Additionally, analyzing these charts helps provide the best parameter settings for improving the algorithm’s efficiency. It also aids in selecting the best model with minimal complexity. These charts indicate the sensitivity of parameters to each other, which can be very useful in decision-making regarding the effects of each parameter. These charts thoroughly evaluate the interaction between different parameters and demonstrate the optimization impact of these parameters. Considering the densely populated population in the chart, it is evident that there is a strong interaction between parameters x_1_ and x_3_, as well as between x_1_ and x_4_. Meanwhile, there is a weak interaction between x_1_ and x_2_, x_2_ and x_3_, and x_2_ and x_4_. The weakest interaction is between x_3_ and x_4_. Parameters with weak interaction between them indicate that a change in one parameter will not significantly affect another parameter. In other words, the impact of each parameter in optimization should be examined separately. Conversely, strong interaction indicates a strong interactive effect between parameters and their simultaneous impact on optimization. When there is a strong interaction between parameters, the optimization process of the model is conducted more harmoniously, considering the interactive effects. Whereas, when a parameter is examined alone, parameter settings may not be as effective. In reality, making correct and principled decisions about parameter tuning and achieving better and more accurate results in optimization requires a precise evaluation of the interaction between parameters and their effects on the genetic algorithm. As portrayed in Fig. 9, the colors represent objective function values, with yellow and blue indicating the highest and lowest fitness values, respectively. Clusters of red dots in certain areas signify optimal points based on the interaction of two independent parameters. Yellow dots denote the best positions for achieving optimal results. The optimal ranges for X_1_ and X_2_ are between 5 to 10 and 2.6 to 4, respectively, while for X_3_ and X_4_, the optimal ranges considering parameter interaction are 115.5 to 118 and 0.1 to 0.6, respectively. Some parameters show minimal interactive effects. This diagram helps pinpoint model strengths and weaknesses and identify optimal points within the genetic algorithm. The color gradient reflects model improvement, shifting from blue to yellow to indicate better optimization, with yellow clusters highlighting high-value algorithmic optimal points. Figure 9 demonstrates a substantial interaction between the parameter pairs (X_1_, X_2_) and (X_2_, X_3_). The red points are concentrated in a yellow area, indicating a strong correlation between X_1_ and X_2_, as well as X_2_ and X_3_. Conversely, for the parameter pairs (X_1_, X_4_) and (X_3_, X_4_), the interaction between the parameters is weaker. The red points are more dispersed in the yellow area, depicting a weaker correlation between (X_1_, X_4_) and (X_3_, X_4_). The red points in the charts represent potential solutions identified by the genetic algorithm. These points are randomly generated during the genetic algorithm's search process and are then assessed based on their fitness to the objective function. This indicates that these points are potential solutions likely to yield the best values for the objective function. It's important to note that the number of red points in the charts depends on the genetic algorithm's settings. Some red points may be situated in a darker area. These points represent potential solutions with lower fitness to the objective function but may still be worth considering. To pinpoint the most accurate optimal point, a more precise parameter search may be necessary. For the parameter pairs (X_1_, X_2_) and (X_2_, X_3_), the optimal points are widely dispersed and do not follow a distinct pattern. This suggests that the relationship between these parameters is more complex, making it challenging to find a single optimal solution. The genetic algorithm is inspired by the process of evolution in nature. In this algorithm, a set of initial solutions is randomly generated. These solutions are then evaluated based on their fitness to the objective function. Solutions with higher fitness are more likely to pair with each other. This process is repeated until an "optimal" solution is found. In these charts, the genetic algorithm has repeatedly altered the parameter values and calculated the objective function for each new combination. Since the genetic algorithm operates randomly, the dispersion of optimal points in the (X_1_, X_2_) and (X_2_, X_3_) charts indicates that the relationship between these parameters is complex, and the optimal values of one parameter significantly depend on the values of the other parameter. Conversely, for the parameter pairs (X_1_, X_4_) and (X_3_, X_4_), the dispersion of optimal points is less compared to the pairs (X_1_, X_2_) and (X_2_, X_3_). (If the optimal points form a straight line or a smooth curve, it indicates a linear or weak relationship between the two parameters). This suggests that the relationship between these parameters is weaker. In other words, while the optimal values of one parameter in the pairs (X_1_, X_2_) and (X_2_, X_3_) significantly depend on the values of the other parameter, this dependency is less for the pairs (X_1_, X_4_) and (X_3_, X_4_). This means that optimal values for X_1_ and X_4_ (or X_3_ and X_4_) can be found relatively independently of each other. The lesser dispersion of optimal points in the (X_1_, X_4_) and (X_3_, X_4_) charts indicates that the algorithm was significantly less sensitive to changes in X_1_ and X_4_ compared to (X_1_ , X_2_ ) and (X_2_ and X_3_)^37,58–60^.

**Figure 10:**
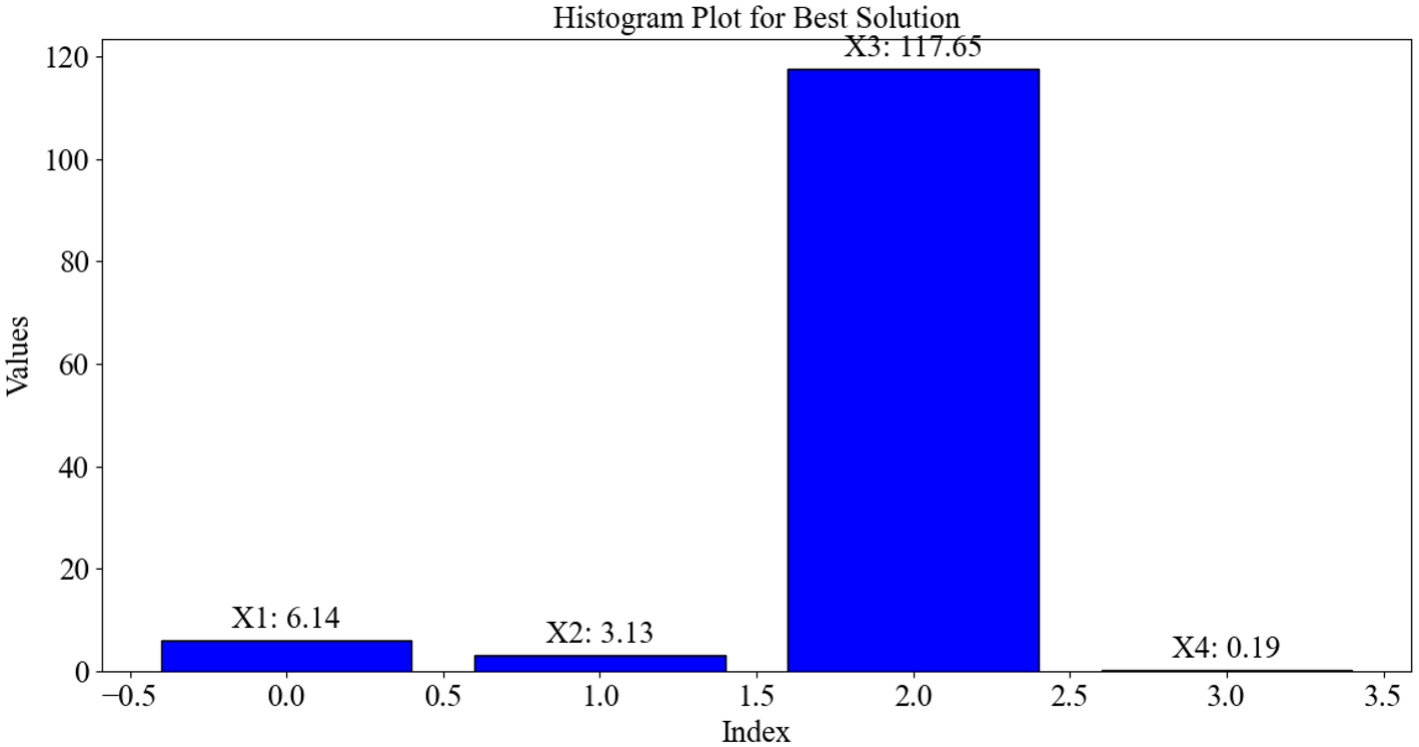
The optimal conditions and values for each parameter determined by GA.

Figure 10 presents the optimal conditions and values for each parameter determined by GA. In the chart, the values of INDEX and VALUE indicate the parameter's position and its optimal value, respectively^61,62^. For instance, parameter x_3_ is positioned at 2 in the chart and has an optimal value of 117.65. In this way, variables are distinguished from each other. According to the chart, the best values for x_1_, x_2_, x_3_, and x_4_ are 6.14 mg L^−1^, 3.13, 117.65 min, and 0.19 g L^−1^, respectively. Indeed, this chart allows decision-makers in the various industrial sectors to gain a better understanding of the performance of the genetic algorithm in the different optimization phases.

Initially, after activating SOLVER, the uncoded formula provided by the R software (Eq. 7), was used to define and specify variables and the objective function. In other words, using Eq. (7), the optimization function was created in a cell, and variables for which optimal values needed to be determined were set up in a column. First, the cell containing the objective function was selected. Then, the goal of the objective function was specified, indicating whether the objective function should reach a specific value, a minimum value, or a maximum value. Next, the variables to be optimized were selected. The necessary constraints for the objective function were specified. Subsequently, the type of SOLVER, such as Simplex LP or GRG Nonlinear, was identified. Then, optimization was performed by the system, and the best variable values were determined in the results. According to the cases mentioned in the maximum amount of the response variable, the values of x_1_, x_2_, x_3,_ and x_4_ parameters were determined to be 5 mg L^−1^, 3, 120 min, and 0.19 g L^−1^ respectively. Therefore, in comparing the results between the SOLVER and GA models, the following outcomes were obtained: The optimization results obtained from the SOLVER and GA models yield slightly different optimal values for the parameters. Specifically, for parameter (x_1_), the SOLVER model suggests an optimal value of 5 mg L^−1^, while the GA model indicates a slightly higher value of 6.14 mg L^−1^. Both models converge on the same optimal value of 3 for parameter (x_2_). However, there is a slight discrepancy in the optimal value for parameter (x_3_), with the SOLVER model suggesting 120 min and the GA model indicating 117.65 min. Nonetheless, both models agree on an optimal value of 0.19 g L^−1^ for parameter (x_4_). Overall, while there are minor differences between the optimization results of the SOLVER and GA models, they generally exhibit a degree of agreement in identifying optimal parameter values. In Table 4, a comparison between different processes in pollutant removal using various artificial intelligence models is provided.

**Table 4:** The application of various artificial intelligence models in evaluating different processes.

Title	Models Employed	Performance Evaluation	Identification of Significant Factors	Optimization Results	References
Thermodynamical and artificial intelligence approaches of H_2_S solubility in *N*-methylpyrrolidone	utilized ANN and GA for predicting H_2_S solubility, achieving precision comparable to thermodynamical modeling	compared GA with experimental and thermodynamic data, achieving sound agreement	identified significant factors for H_2_S solubility using GA	employed GA to optimize the design of neural network models for predicting H_2_S solubility, enhancing the effectiveness of the forecasting scheme	^63^
Efficient prediction of water vapor adsorption capacity in porous metal–organic framework materials: ANN and ANFIS modeling	employed ANN and ANFIS to predict water vapor adsorption in MOFs, outperforming experimental data with MSE of 0.005 and 0.002, respectively	Evaluated ANN and ANFIS performance with MSE values of 0.005 and 0.002, respectively	identified surface area, pore volume, and pore diameter as significant factors for water vapor adsorption in MOFs	utilized ANN and ANFIS methods to predict water vapor adsorption capacity in MOFs, providing a robust tool for quick screening of appropriate porous materials for dehumidification	^64^
Thermal degradation evaluation of polyethylene terephthalate microplastics: Insights from kinetics and machine learning algorithms using non-isoconversional TGA data	utilized SVM, DTM, RFM, and ANN to predict thermal behavior of PET MPs, with RFM showing the best performance (R^2^ = 0.999)	utilized various metrics to evaluate different models, with RFM exhibiting the lowest error metrics	identified various parameters influencing thermal behavior of PET MPs	optimized the thermal degradation analysis of PET MPs using various machine learning algorithms, including SVM, DTM, RFM, and ANN, with RFM consistently outperforming other models and providing reliable predictions	^33^
Comparison of modeling, optimization, and prediction of important parameters in the adsorption of cefixime onto sol–gel derived carbon aerogel and modified with nickel using ANN, RSM, GA, and SOLVER methods	employed ANN, RSM, GA, and SOLVER for optimizing Cefixime adsorption onto SGCAN, with ANN exhibiting superior performance (R^2^ = 0.98)	assessed ANN and SVR performance, with SVR demonstrating higher R^2^ (0.98), and lower MAE (1.54) and RMSE (3.91)	identified pH, time, and catalyst amount as significant factors for Cefixime adsorption onto SGCAN	optimized Cefixime adsorption using GA, achieving the highest adsorption capacity of 52 mg g^−1^	^58^
Hybrid approach based on response surface methodology and artificial neural networks coupled with genetic algorithm (RSM-GA-ANN) for the Prediction and optimization for the Photodegradation of dye using nano ZnO anchored glass fiber under solar light irradiation	used RSM-ANN and RSM-(GA-ANN) models for predicting AB 10B dye degradation, with RSM-(GA-ANN) showing the highest R^2^ (0.9669)	evaluated RSM-ANN and RSM-(GA-ANN) models, with RSM-(GA-ANN) showing the highest R^2^ (0.9669)	identified influential factors for AB 10B dye degradation	optimized AB 10B dye degradation, with RSM-(GA-ANN) model providing the best results	^37^
Experimental design, RSM and ANN modeling of tetracycline photocatalytic degradation using LDH@CN	employed CCD and ANN for predicting TC photocatalytic degradation, with ANN demonstrating higher predictive capability	assessed ANN model's performance, demonstrating higher predictive capability	identified parameters affecting TC photocatalytic degradation	optimized TC photocatalytic degradation using ANN, achieving high decomposition efficiency of 96%	^38^
Cefixime Removal via WO_3_/Co-ZIF Nanocomposite Using Machine Learning Methods	utilized SVR, GA, ANN, SOLVER, and RSM for optimizing Cefixime removal, with SVR achieving the best results (R^2^ = 0.98)	evaluated different models based on MAE, RMSE, and R^2^ Score, with SVR selected as the best model	identified significant factors impacting Cefixime removal	optimized Cefixime removal, with GA determining the optimum values for various parameters	The present study

## Conclusion

This research was aimed at the application of the WO_3_/Co-ZIF photocatalytic process for the removal of Cefixime from the aqueous solutions. Artificial intelligence models were utilized in this study for data prediction and optimization. It was observed artificial intelligence models exhibit high efficiency in interpreting the data. Based on the obtained results, these models due to their high prediction accuracy, rapid attainment of optimal conditions, reduction in the need for extensive experiments, and analysis of complex relationships between various variables can be highly effective and efficient in interpreting and optimizing photocatalytic processes. The SVR model was employed as a suitable model for data interpretation due to its lower error compared to the ANN model, and the prediction of response results was carried out based on this model. pH was identified as the most crucial parameter in data prediction by the SVR model. According to the GA, the most important parameters that had significant interactions leading to optimal results included the interaction between the initial concentration of Cefixime with time and the initial concentration of Cefixime with the catalyst amount. Based on GA, the optimal conditions for Cefixime removal from the aqueous solutions were determined to be an initial concentration of Cefixime of 6.14 mg L^−1^, pH of 3.13, a time of 117.65 min, and a catalyst amount of 0.19 g L^−1^. The findings of this research can significantly contribute to the advancement of intelligent decision-making and optimization in industrial and environmental processes, thereby enhancing environmental sustainability.

### Supplementary Information

Supplementary Information.

## Data Availability

The datasets used during the current study available from the corresponding author on reasonable request.
