# Supplementary material for: Cefixime removal via WO3/Co-ZIF nanocomposite using machine learning methods
Source: Sci Rep. 2024 Jun 15;14:13840. doi: 10.1038/s41598-024-64790-2 (PMC11180210; doi:10.1038/s41598-024-64790-2)
Supplement: Supplementary file 1 — Supplementary Information. [file 41598_2024_64790_MOESM1_ESM.docx]

**Supporting Information for**

**Cefixime Removal via WO3/Co-ZIF Nanocomposite Using Machine Learning Methods**

Amir Sheikhmohammadi^1^, Hassan Alamgholiloo^1^, Mohammad Golaki^2^, Parsa Khakzad^1^, Esrafil Asgari^3,*^, Faezeh Rahimlu^1^

^1^Department of Environmental Health Engineering, School of Health, Khoy University of Medical Sciences, Khoy, Iran

^2^Department of Environmental Health Engineering, School of Health, Shiraz University of Medical Sciences, Shiraz, Iran

^3^ Department of Environmental Health Engineering, School of Public Health, Zanjan University of Medical Sciences, Zanjan, Iran

*Corresponding author: E. Asgari ( E-mail addresses: asgari.esrafil@zums.ac.ir)

Design based on RSM model

The objective of designing based on this model is to aid in analyzing the simultaneous effects of the various variables on a response. After the design matrix was generated using CCD in the R software, the data and responses were fitted with three RSM models (Factorial model, Quadratic model and Factorial-quadratic model). These three models were utilized for statistical analysis and designing response surface models, these models are included in the supplementary file.

1. Factorial model, examining main effects and interaction effects between variables.

2. Quadratic model, incorporating quadratic (square) effects of variables on the response.

3. Factorial-quadratic model, investigating main effects, interaction effects, and quadratic effects of variables.

The criteria considered for comparing the three models included statistical tests and the results of ANOVA analysis. These tests and analyses consisted of the following:

1. R-squared (R^2^): Indicates the model's ability to explain all variations concerning independent variables in the response (dependent variable). The closer it is to 1, the better the model aligns with the data.

2. AIC (Akaike Information Criterion): Represents the relationship between model simplicity and its ability to explain the data. A model with a lower AIC has greater explanatory power.

3. Lack of fit: Illustrates the model's incompatibility with real data. When the model fits well with the data, the lack of fit is insignificant, indicating minimal difference between model predictions and real data.

4. F-statistic and p-value: Essentially signify the significance and alignment of the model with the data. A higher F-statistic value and a significant p-value indicate better model alignment with the data.

5. Residual Sum of Squares (RSS): Used to measure the level of agreement between the model and experimental data. A lower sum of squares of differences between model predictions and actual data values indicates a better fit of the model to the provided data.

**Table S1** The predicted results of response for the different models

| Run | X_1_ | X_2_ | X_3_ | X_4_ | Actual Removal (%) | RSM Removal (%) | ANN Removal (%) | SVM Removal  (%) |
| --- | --- | --- | --- | --- | --- | --- | --- | --- |
| 1 | 12.5 | 6 | 62.5 | 0.12 | 32.8 | 34 | 34.34 | 35.2 |
| 2 | 16.25 | 4.5 | 33.75 | 0.085 | 40 | 39.79 | 39.88 | 36.55 |
| 3 | 8.75 | 7.5 | 33.75 | 0.155 | 20.2 | 18.4 | 19.93 | 18.95 |
| 4 | 12.5 | 6 | 62.5 | 0.12 | 33.7 | 34 | 34.34 | 35.2 |
| 5 | 16.25 | 7.5 | 91.25 | 0.085 | 18 | 14.8 | 17.69 | 16.8 |
| 6 | 16.25 | 4.5 | 91.25 | 0.085 | 55 | 55.8 | 56.12 | 53.44 |
| 7 | 16.25 | 7.5 | 33.75 | 0.085 | 4 | 16.55 | 9.89 | 6.5 |
| 8 | 8.75 | 7.5 | 33.75 | 0.085 | 8 | 20.59 | 9.26 | 8.5 |
| 9 | 16.25 | 7.5 | 91.25 | 0.155 | 30 | 30.3 | 31.25 | 29.49 |
| 10 | 12.5 | 6 | 62.5 | 0.12 | 34.8 | 34 | 34.34 | 35.2 |
| 11 | 8.75 | 4.5 | 91.25 | 0.085 | 58 | 60.4 | 59 | 57.79 |
| 12 | 8.75 | 7.5 | 91.25 | 0.085 | 20.1 | 19.4 | 20.1 | 21.19 |
| 13 | 12.5 | 6 | 62.5 | 0.12 | 35.1 | 34 | 34.34 | 35.2 |
| 14 | 8.75 | 4.5 | 33.75 | 0.155 | 54 | 55.3 | 60.64 | 53.5 |
| 15 | 12.5 | 6 | 62.5 | 0.12 | 32 | 34 | 34.34 | 35.2 |
| 16 | 12.5 | 6 | 62.5 | 0.12 | 32.8 | 34 | 34.34 | 35.2 |
| 17 | 16.25 | 7.5 | 33.75 | 0.155 | 16 | 13.4 | 15.42 | 12.6 |
| 18 | 8.75 | 4.5 | 91.25 | 0.155 | 90 | 89.2 | 90.44 | 90.1 |
| 19 | 16.25 | 4.5 | 91.25 | 0.155 | 87 | 84.1 | 86.5 | 86.5 |
| 20 | 8.75 | 7.5 | 91.25 | 0.155 | 35 | 34 | 34.34 | 35.2 |
| 21 | 8.75 | 4.5 | 33.75 | 0.085 | 45 | 44.4 | 45.2 | 40.9 |
| 22 | 16.25 | 4.5 | 33.75 | 0.155 | 49 | 50.7 | 50.19 | 49.02 |
| 23 | 12.5 | 6 | 62.5 | 0.12 | 34.7 | 34 | 34.34 | 35.2 |
| 24 | 12.5 | 6 | 62.5 | 0.19 | 47.6 | 47.2 | 48.7 | 47.85 |
| 25 | 12.5 | 6 | 62.5 | 0.12 | 32.1 | 34 | 34.34 | 35.2 |
| 26 | 12.5 | 6 | 62.5 | 0.12 | 35 | 34 | 35.38 | 33.8 |
| 27 | 5 | 6 | 62.5 | 0.12 | 40 | 38.4 | 42.34 | 39.5 |
| 28 | 12.5 | 6 | 62.5 | 0.12 | 31.8 | 34 | 34.34 | 35.2 |
| 29 | 12.5 | 6 | 62.5 | 0.12 | 33 | 34 | 34.34 | 35.2 |
| 30 | 12.5 | 6 | 5 | 0.12 | 20 | 25.3 | 20.2 | 19.31 |
| 31 | 12.5 | 6 | 62.5 | 0.12 | 36.1 | 34 | 34.34 | 35.2 |
| 32 | 12.5 | 6 | 120 | 0.12 | 56.5 | 58.1 | 57.3 | 57.08 |
| 33 | 12.5 | 3 | 62.5 | 0.12 | 92.5 | 91.2 | 92.47 | 92.2 |
| 34 | 12.5 | 6 | 62.5 | 0.12 | 34.7 | 34 | 34.34 | 35.2 |
| 35 | 20 | 6 | 62.5 | 0.12 | 31 | 29.3 | 32.1 | 30.84 |
| 36 | 12.5 | 6 | 62.5 | 0.12 | 35.4 | 34 | 34.34 | 35.2 |
| 37 | 12.5 | 6 | 62.5 | 0.05 | 24 | 20.4 | 19.38 | 22.5 |
| 38 | 12.5 | 6 | 62.5 | 0.12 | 34.9 | 34 | 34.34 | 35.2 |
| 39 | 12.5 | 9 | 62.5 | 0.12 | 11.3 | 13.1 | 11.75 | 12.1 |

**Table S2.** Regression analysis for uncoded date

| Model term | Coefficient estimate | Std. error | t-Value | p-Value |
| --- | --- | --- | --- | --- |
| Intercept | 1.115e+02 | 1.326e+01 | 8.411 | 2.87e-09 |
| x_1_ | -6.056e-01 | 1.334e-01 | -4.540 | 9.09e-05 |
| x_2_ | -2.352e+01 | 2.830e+00 | -8.312 | 3.66e-09 |
| x_3_ | 6.034e-02 | 1.305e-01 | 0.462 | 0.64737 |
| x_4_ | 2.914e+02 | 8.095e+01 | 3.600 | 0.00117 |
| x_2_^^2^ | 2.026e+00 | 1.891e-01 | 10.715 | 1.35e-11 |
| x_3_^^2^ | 2.369e-03 | 5.146e-04 | 4.603 | 7.64e-05 |
| x_2_:x_3_ | -9.986e-02 | 1.421e-02 | -7.029 | 9.90e-08 |
| x_2_:x_4_ | -6.274e+01 | 1.167e+01 | -5.377 | 8.91e-06 |
| x_3_:x_4_ | 4.379e+00 | 6.088e-01 | 7.193 | 6.43e-08 |


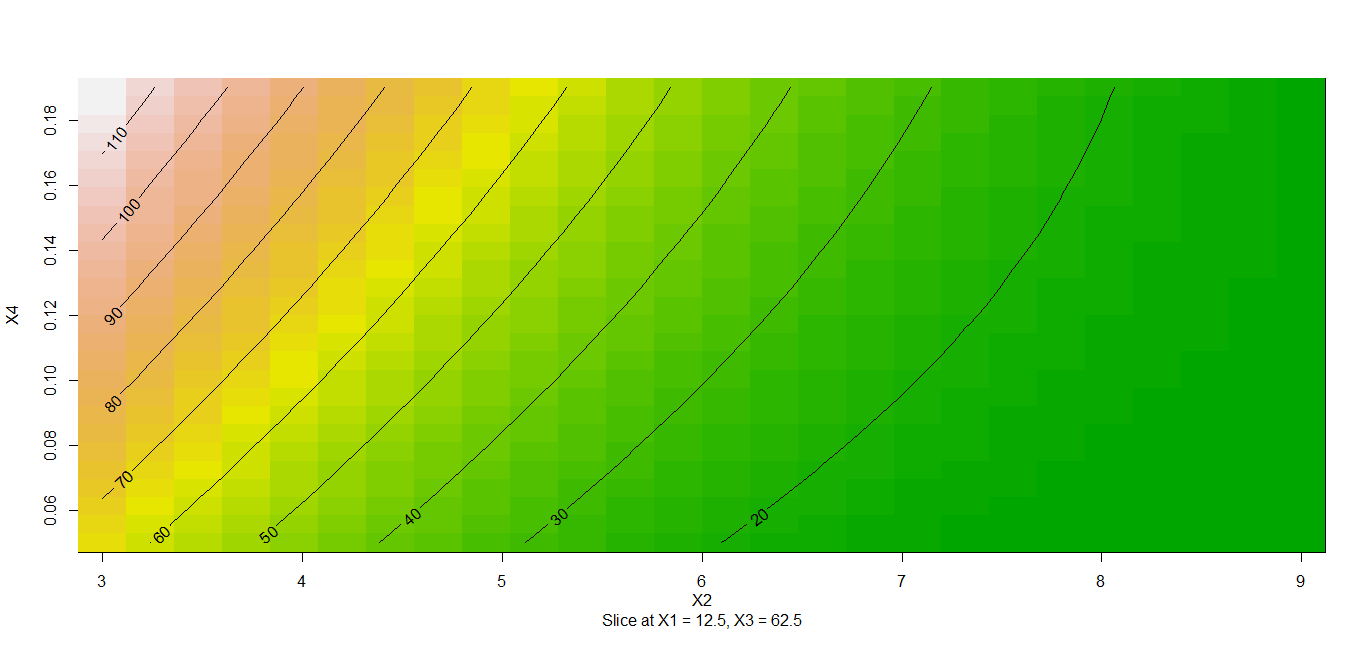

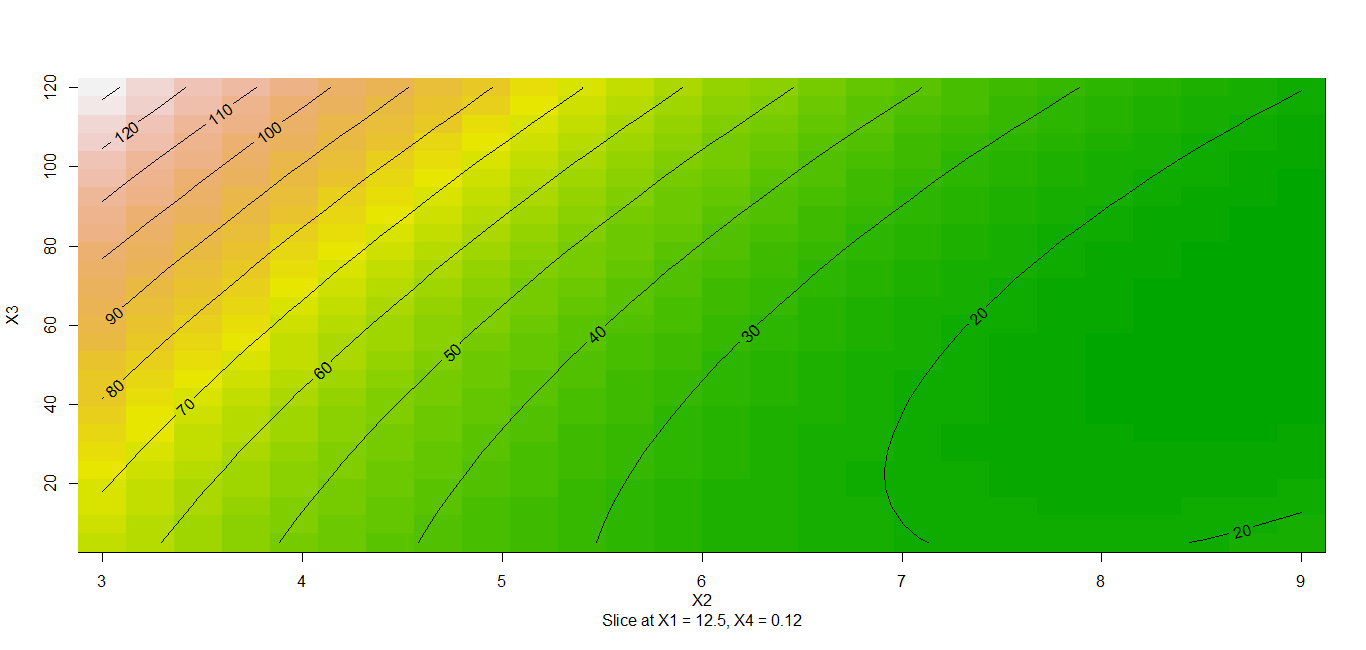


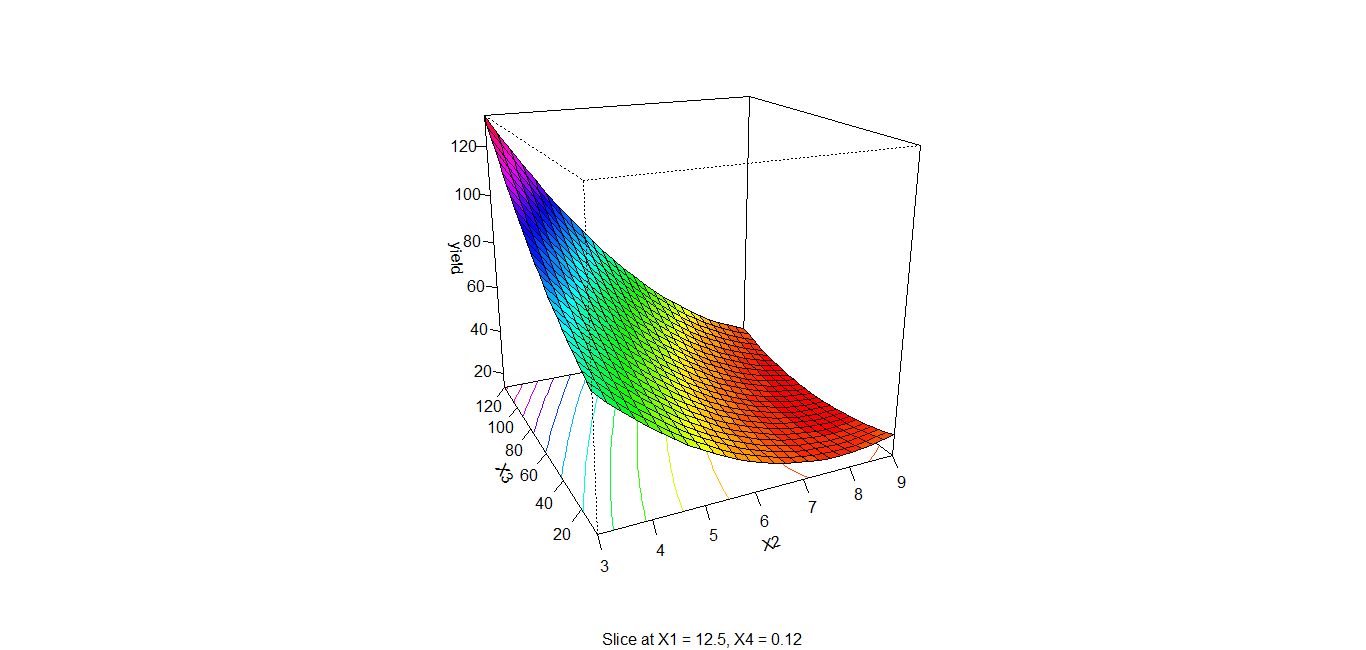

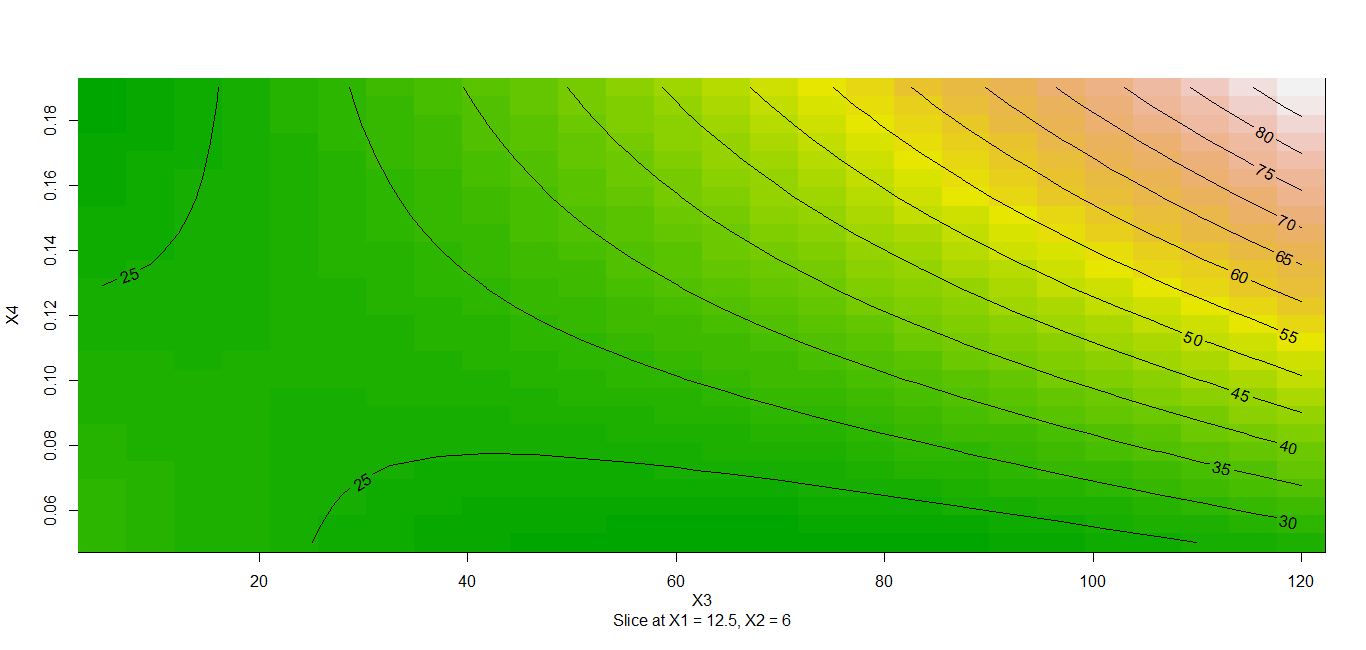


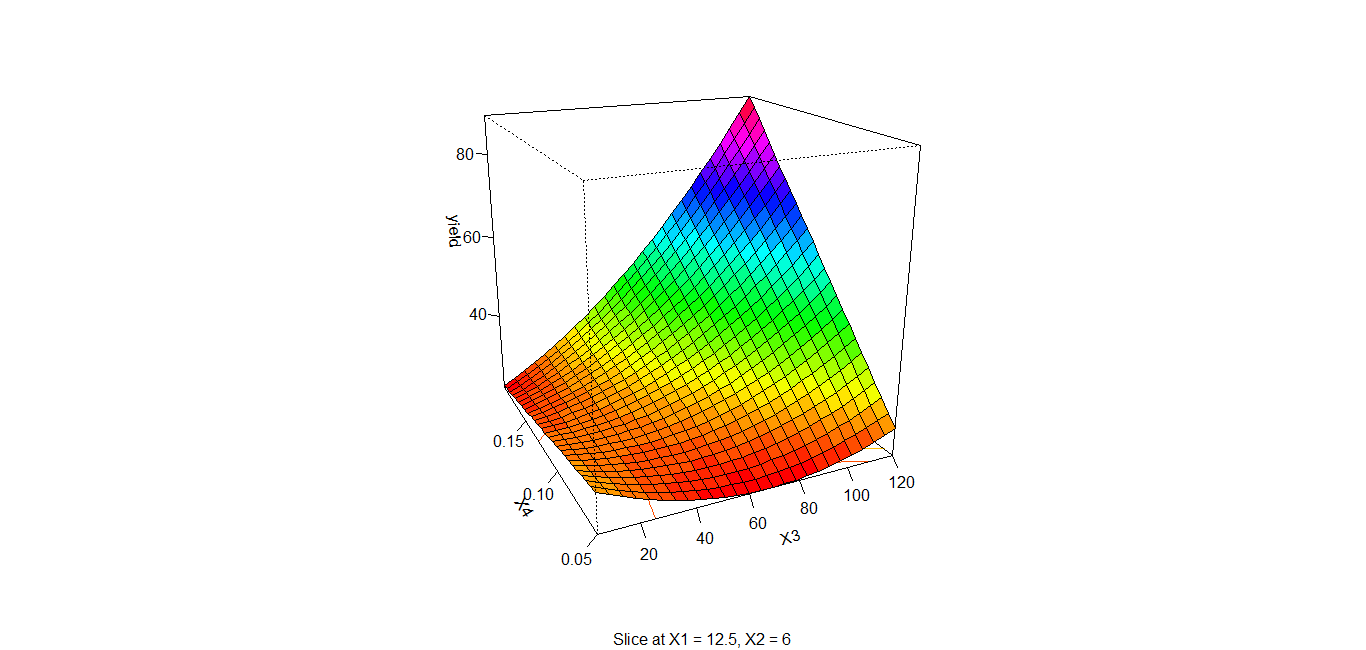

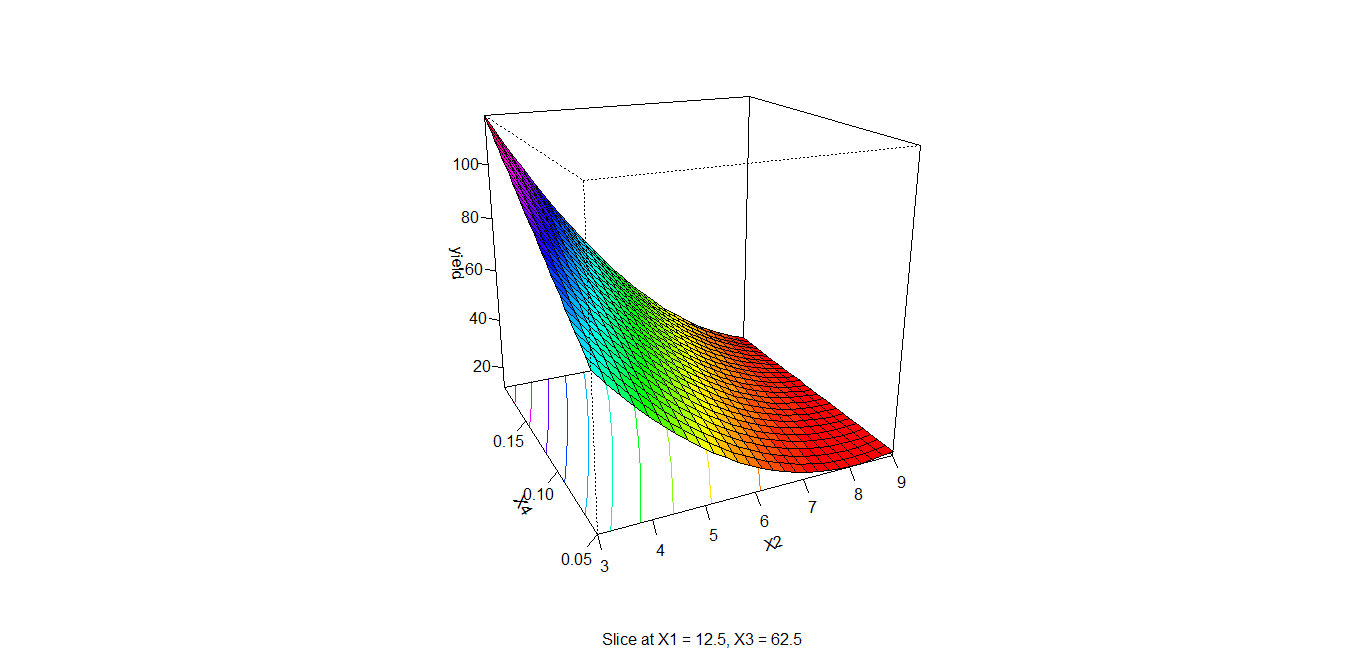


**Fig. S2.** Influence of the interaction between variables on the response (dependent variable) using contour and perspective plots.
